# Mesenchymal stem cell-derived small extracellular vesicles mitigate oxidative stress-induced senescence in endothelial cells via regulation of miR-146a/Src

**DOI:** 10.1038/s41392-021-00765-3

**Published:** 2021-10-22

**Authors:** Xian Xiao, Meiqian Xu, Hongliang Yu, Liping Wang, Xiaoxia Li, Janusz Rak, Shihua Wang, Robert Chunhua Zhao

**Affiliations:** 1 Institute of Basic Medical Sciences Chinese Academy of Medical Sciences, School of Basic Medicine Peking Union Medical College, Beijing, China; 2 Department of Genetics and Cell Biology, Basic medical college, Qingdao University, 308 Ningxia Road, 266071 Qingdao, China; 3 Research Institute of the McGill University Health Centre, Glen Site, McGill University, Montreal, QC H4A 3J1 Canada; 4 Department of Cell Biology, School of Life Sciences, Shanghai University, 200444 Shanghai, China

**Keywords:** Senescence, Mesenchymal stem cells

## Abstract

Senescent endothelial cells (ECs) could impair the integrity of the blood vessel endothelium, leading to vascular aging and a series of diseases, such as atherosclerosis, diabetes. Preventing or mitigating EC senescence might serve as a promising therapeutic paradigm for these diseases. Recent studies showed that small extracellular vesicles (sEV) have the potential to transfer bioactive molecules into recipient cells and induce phenotypic changes. Since mesenchymal stem cells (MSCs) have long been postulated as an important source cell in regenerative medicine, herein we investigated the role and mechanism of MSC-derived sEV (MSC-sEV) on EC senescence. In vitro results showed that MSC-sEV reduced senescent biomarkers, decreased senescence-associated secretory phenotype (SASP), rescued angiogenesis, migration and other dysfunctions in senescent EC induced by oxidative stress. In the In vivo natural aging and type-2 diabetes mouse wound-healing models (both of which have senescent ECs), MSC-sEV promoted wound closure and new blood vessel formation. Mechanically, miRNA microarray showed that miR-146a was highly expressed in MSC-sEV and also upregulated in EC after MSC-sEV treatment. miR-146a inhibitors abolished the stimulatory effects of MSC-sEV on senescence. Moreover, we found miR-146a could suppress Src phosphorylation and downstream targets VE-cadherin and Caveolin-1. Collectively, our data indicate that MSC-sEV mitigated endothelial cell senescence and stimulate angiogenesis through miR-146a/Src.

## Introduction

Aging induced vascular dysfunctions play a critical role in the pathogenesis of a variety of age-related diseases, such as delayed wound healing, heart failure, diabetes, Alzheimer’s disease, and kidney diseases^1,2^. In demographic research, vascular diseases are the leading causes of severe long-term impairment and mortality in older adults^3,4^. Therapeutic strategies to reduce vascular diseases by preventing or eliminating vascular senescence are still required, despite years of intensive research^5^. Senescent endothelial cells (ECs) exhibit major changes in gene expression, cell replication and morphology, impair the integrity of the endothelium in vascular by influencing the endothelium’s regenerative and angiogenic capacities, reactivity and pathogenic progression, contributing to vascular aging diseases^6,7^. A range of factors can cause endothelial senescence; among them, oxidative stress has a major role^8^. H_2_O_2_ and high glucose are factors contributing to ECs senescence as oxidative stresses^9,10^. Ming-Hui Zou showed that H_2_O_2_ could trigger HUVECs and human aortic smooth muscle cells senescence through Oct4A upregulation^11^. H_2_O_2_-induced EC senescence provokes a DNA-damage response, which results in activation of p53 and p16, the important cell cycle regulatory pathways^12^. High-glucose induced HUVECs senescence through decreasing the expression level of SIRT3, and increased SA-gal expression and damaged the tube forming ability^9^. Oxidative stress participates in the pathogenesis of vascular abnormalities in diabetes which can induce premature senescence via DNA damage, and Streptozotocin(STZ)-induced diabetes can cause ECs senescence^13^. However, the potential interventions to prevent ECs senescence for the prevention of vascular pathologies are unclear.

Mesenchymal stem cells (MSCs), identified as multipotent stromal cells, have multi-lineage differentiation ability and immunosuppressive properties^14^ (Supplementary Fig. 1B–F). They can be derived from several sources, including the umbilical cord, bone marrow, or fat tissue, making them ideal for immunomodulation and regeneration as a promising candidate cell type^15^. Increasing evidence indicates that these features of MSCs are linked to their paracrine activity and extracellular vehicles (EVs) secretion^16^. Small extracellular vesicles (sEV) are extracellular vesicles generated by fusion with the cellular membrane of multivesicular bodies. They are between 30–150 nm in diameter and contain abundant functional components such as proteins and microRNAs (miRNAs)^17^. sEV have emerged as a complex means for a variety of cellular processes to be modulated^18^. sEV derived from MSCs have previously been documented to elicit similar therapeutic effects to their parent MSCs^19^. More specifically, sEV released by MSCs, such as low immunogenicity, easy storage, and high biosafety, have striking advantages over whole-cell therapy^16^. sEV derived from MSC have been shown to have great promise in anti-inflammation and injury repair^20,21^ and are commonly studied as a nanotherapeutic agent for stroke^22^ and wound-healing treatment^23^. Thus, for certain diseases, MSC-sEV have been described as highly promising cell-free therapeutic agents. Nonetheless, the curative effects and mechanisms of the action of MSC-sEV on senescence are poorly understood, to the best of our knowledge, particularly on EC senescence.

We intended to investigate the effect of MSC-sEV on oxidative stress-induced HUVECs senescence in vitro, and naturally aged and diabetic mice wound-healing model in vivo. Moreover, we investigated the underlying molecular mechanism by using miRNA sequencing and phospho-kinase antibody array. Our results suggested that MSC-sEV can act as a nanotherapeutic agent via miR-146/Src pathway to prevent oxidative stress-induced EC senescence.

## Results

### Characterization of MSC-sEV and their active internalization by HUVECs

**Fig. 1:**
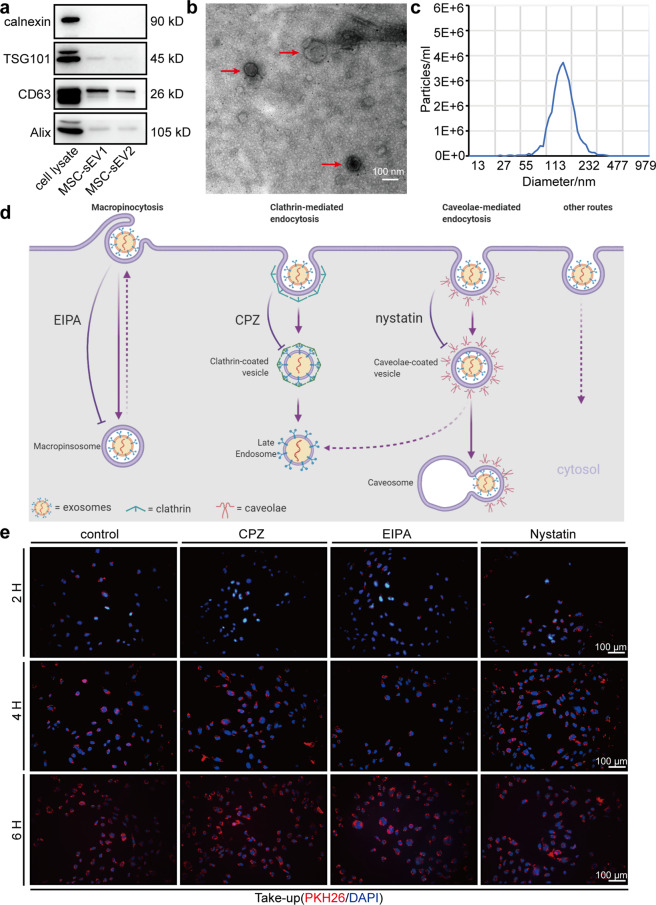
Characterization of MSC-sEV and their active internalization by HUVECs. **a** Representative images of western blot analysis showing the biomarkers of sEV, including TSG101, CD63, and Alix. Calnexin was used as a negative control (MSC-sEV1, 2.1 mg protein/mL MSC-sEV; MSC-sEV2, 0.21 mg protein/mL MSC-sEV). **b** Transmission electron microscopic images of MSC-sEV (scale bar, 100 nm). **c** Nanoparticles tracking analysis reveals the particle distribution of exosomes in various sizes. Within every nanometer diameter sets, the value of the ordinate represents the mean particle number. **d** Schematic illustration of the different types of endocytosis and their respective inhibitor (Created with BioRender.com). **e** Uptake of MSC-sEV by HUVEC pretreated with signal inhibitors chlorpromazine (CPZ), nystatin, or 5-(N-ethyl-N-isopropyl) amiloride (EIPA) for 1 h. Cells were incubated with the indicated inhibitors for 1 h before and during the incubation with MSC-sEV. HUVECs incubated with 200 ng/μL PKH26-labeled MSC-sEV for the indicated times, and uptake of MSC-sEV was determined by fluorescence microscopy (scale bar, 100 μm)

sEV were isolated from supernatant of MSCs using ultracentrifugation and characterized by western blot, transmission electron microscopy (TEM) and nanoparticle tracking analysis (NTA). sEV-associated protein markers Alix and CD63(transmembrane/lipid-bound protein) and TSG101(cytosolic protein) were enriched in MSC-sEV and the negative protein marker calnexin (an endoplasmic reticulum marker) was not found in MSC-sEV compared to MSC lysate (Fig. 1a). Under TEM, MSC-sEV exhibited classic cup-shaped or sphere-shaped morphology (Fig. 1b). According to NTA, the distribution curve of the particle size of MSC-sEV was between 55 and 200 nm (Fig. 1c). To investigate whether MSC-sEV could be internalized by ECs, we used HUVECs as a cellular model. Supplementary Fig. 1a showed immunofluorescence staining of VWF and CD31 (two typical endothelial markers) in HUVECs. MSC-sEV were labeled with PKH26 and added to HUVECs at a final concentration of 200 ng/μL in the medium. sEV incorporation was observed 2 h after treatment and accumulated in HUVECs over time (Fig. 1e). It has been reported that EVs could be taken up via a variety of endocytic pathways, including macropinocytosis, CME, caveolin-mediated endocytosis, and clathrin- and caveolin-independent endocytosis^24^. Fig. 1d is a schematic illustration of the different types of endocytosis and their respective inhibitor. Treatment with an inhibitor did not reduce the internalization of PKH26-labeled sEV (Fig. 1e, Supplementary Fig. 1g, h), suggesting that MSC-sEV may gain entry into HUVECs via more than one route. Collectively, we characterized MSC-sEV and showed that they are actively incorporated in vitro by HUVECs via multiple routes.

### MSC-sEV mitigated senescence in HUVECs in vitro

**Fig. 2:**
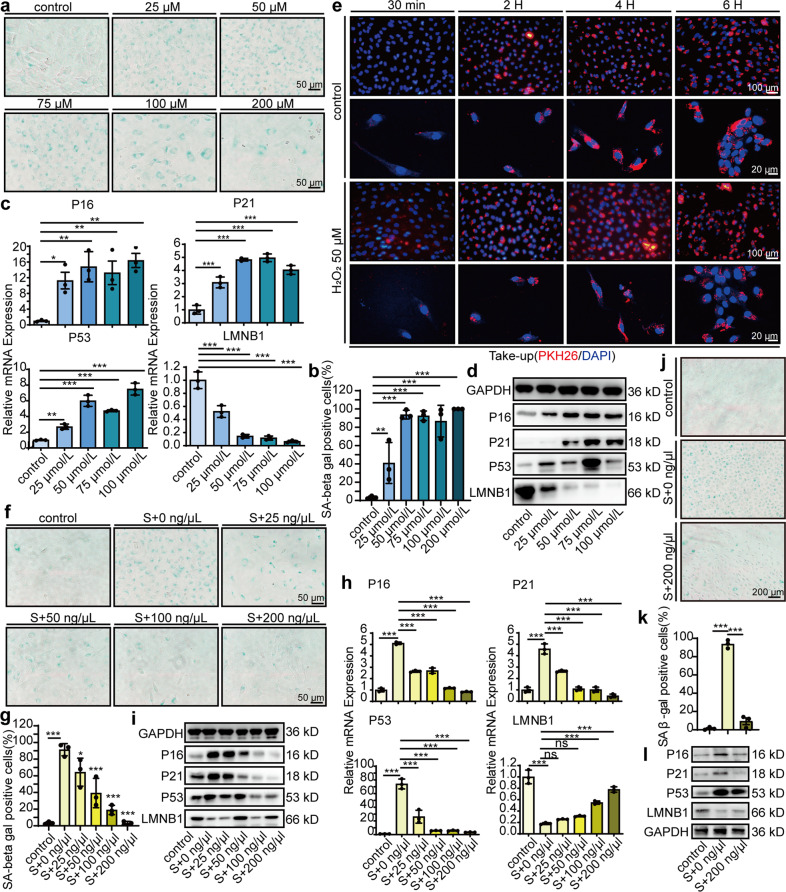
MSC-sEV mitigated senescence in HUVECs in vitro. Cultures exposed to H_2_O_2_ (25 μM, 50 μM, 75 μM, 100 μM, 200 μM, 2 h) or PBS (control) as showing in the figure. **a** Representative images of senescence-associated β-galactosidase (SA β-gal) staining (scale bar, 50 μm). **b** Quantitation of SA β-gal-positive cells in HUVEC. **c** Quantitative real-time PCR (qRT-PCR) of the senescence markers P16, P21, P53, and LMNB1 mRNA in HUVEC after 24 h exposure to H_2_O_2_. *n* = 3, *p < 0.05, **p < 0.01, ***p < 0.001. **d** Representative images of western blot analysis showing the senescence markers P16, P21, P53, and LMNB1 in HUVEC after 48 h of exposure to H_2_O_2_. **e** Representative images of MSC-sEV uptake by HUVEC pretreated with or without H_2_O_2_ (50 μM, 2 h). HUVECs incubated with 200 ng/μL PKH26-labeled MSC-sEV for the indicated times, and uptake of MSC-sEV was determined by fluorescence microscopy (scale bar, 100 μm) and fluorescence confocal microscopy (scale bar, 20 μm). Next, HUVECs were incubated with 0 ng/μL, 25 ng/μL, 50 ng/μL, 100 ng/μL and 200 ng/μL MSC-sEV for 24 h (**c**) and 48 h (**a**, **d**) after pretreated with H_2_O_2_ (50 μM, 2 h). **f** Representative images of SA β-gal staining in HUVECs (scale bar, 50 μm). **g** Quantitation of SA β-gal-positive cells in HUVEC. *n* = 3, **p < 0.01, ***p < 0.001. **h** qRT-PCR of the senescence markers P16, P21, P53, and LMNB1 mRNA in HUVEC. *n* = 3, *p < 0.05, **p < 0.01, ***p < 0.001. **i** Representative images of western blot analysis showing the change of senescence markers P16, P21, P53, and LMNB1 in HUVECs. **j** Representative images of SA β-gal staining in high-glucose-induced senescent HUVECs (scale bar, 200 μm). **k** Quantitation of SA β-gal-positive cells in high-glucose-induced senescent HUVEC. *n* = 3, ***p < 0.001. **l** Representative images of western blot analysis showing the change of senescence markers P16, P21, P53, and LMNB1 in high-glucose-induced senescent HUVEC

H_2_O_2_ has been reported to trigger premature senescence by increasing oxidative stress^11^. To investigate the effect of MSC-sEV on senescence, we first established a proper H_2_O_2_-induced HUVEC senescence model. HUVECs were exposed to different concentrations of H_2_O_2_ (25 μM, 50 μM, 75 μM, 100 μM, 200 μM) for 2 h and several well-established senescence biomarkers were analyzed. H_2_O_2_ exposure increased the percentage of senescence-associated β-galactosidase- (SA β-gal) positive cells with senescence-related morphological transformations: enlarged and flat appearance (Fig. 2a, b). qRT-PCR (Fig. 2c) and western blot (Fig. 2d) showed that H_2_O_2_ markedly elevated senescence markers p16, p21 and p53, and significantly decreased nuclear morphology marker LaminB1. The effect of H_2_O_2_ on HUVECs senescence seemed to be concentration dependent; however, when exceeding 50 μM, the number of adherent cells reduced. Therefore, we chose 50 μM H_2_O_2_ in the following experiments. To investigate whether senescence affects uptake of MSC-sEV, we compared the intensity of PKH26 fluorescence in control and senescent HUVECs. No significant differences were observed (Fig. 2e, Supplementary Fig. 1i). These results suggest that senescent HUVECs remained the capacity to uptake MSC-sEV.

Senescent HUVECs were then treated with different concentrations of MSC-sEV (25, 50, 100, 200 ng/μL). MSC-sEV decreased the percentage of SA β-gal positive cells and recovered senescence-related morphological transformations (Fig. 2f, g). qRT-PCR and western blot showed that MSC-sEV markedly reduced the oxidant-induced elevation of senescence markers p16, p21, p53 and significantly increased nuclear morphology marker LaminB1 at both mRNA (Fig. 2h) and protein level (Fig. 2i). When used at 200 ng/μL, MSC-sEV almost completely recovered HUVEC from senescent state in terms of p16, p21, p53, and LaminB1 expression.

High glucose has also been reported to exerted a pivotal role in senescence through oxidative stress^9^. To confirm the senescence reducing effects of MSC-sEV, we induced HUVECs senescence by high glucose and found similar protective effects of MSC-sEV (Fig. 2–l).

**Fig. 3:**
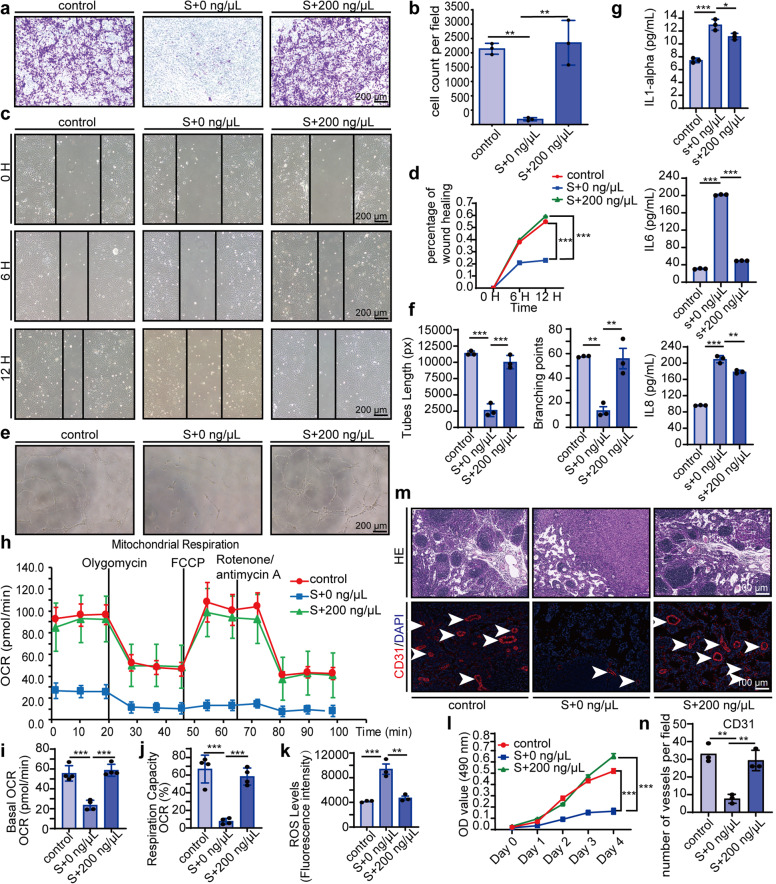
MSC-sEV rescued senescent HUVECs functions. HUVECs were incubated with (s + 200 ng/μL) or without (s + 0 ng/μL) MSC-sEV for 48 h after pretreated with H_2_O_2_ (50 μM, 2 h) compared with normal HUVEC (control). **a** Representative images of transwell migration assays of HUVECs (scale bar, 200 μm). **b** Quantitation of transwell migration assays of HUVECs. *n* = 3, **p < 0.01, ***p < 0.001. **c** Representative images wound-healing assay showing the migratory abilities of HUVECs and the image were taken at the indicated times (scale bar, 200 μm). **d** Quantitation of wound-healing assay at 12 h. *n* = 3, ***p < 0.001. **e** Representative images of in vitro angiogenesis assay (scale bar, 200 μm). **f** Quantitation of mean tube lengths and branching points in the HUVEC network. *n* = 3, **p < 0.01, ***p < 0.001. **g** Quantitation of IL-1 alpha, IL-6, and IL-8 released by HUVEC was detected through ELISA. *n* = 3, *p < 0.05, **p < 0.01, ***p < 0.001. **h** Mitochondrial respiratory capacity measured by O_2_ consumption rate (OCR) using a Seahorse analyzer. **i** Quantitation of basal OCR. *n* = 3, ***p < 0.001. **j** Quantitation of respiration capacity. *n* = 3, ***p < 0.001. **k** Quantitation of the ROS level of HUVEC measured by fluorometric intracellular ROS Kit on a flow cytometer. *n* = 3, **p < 0.01. **l** Quantitation of HUVEC proliferation evaluated by MTS kits with the OD value on Day 0, Day 1, Day 2, Day 3, and Day 4. *n* = 4, **p < 0.01, ***p < 0.001. **m** Representative images of hematoxylin staining and CD31 (red) and DAPI (blue) immunofluorescence staining in paraffin-embedded sections of Matrigel plugs. **n** Quantitation of tubes formed in vivo. Arrows indicate tubes formed in vivo. *n* = 3, **p < 0.01

We then examined the effects of MSC-sEV on HUVECs functions. MSC-sEV remarkably increased senescent HUVEC migration as evidenced by the transwell migration assay (Fig. 3a, b) and scratch wound assay (Fig. 3c, d). Senescent HUVECs treated with MSC-sEV recovered tube formation in vitro (Fig. 3e, f) and blood vessel formation in Matrigel plugs in vivo (Fig. 3m, n). We also tested proinflammatory cytokines IL-1alpha, IL-6, and IL-8, which are markers of the senescence-associated secretor phenotype (SASP)^25^ and found MSC-sEV suppressed IL-6 and IL-8 expression in senescent HUVECs (Fig. 3g). Additionally, we examined mitochondrial respiration and reactive oxygen species (ROS) levels. Senescent HUVECs had reduced basal mitochondrial O_2_ consumption rate (OCR) and mitochondrial respiration capacity, and increased ROS levels, which were significantly rescued by MSC-sEV (Fig. 3h, j, k). Moreover, MSC-sEV promoted senescent HUVECs proliferation (Fig. 3l). Taken together, these results suggest that MSC-sEV could prevent oxidative stress-induced senescence in HUVECs.

### MSC-sEV promoted wound closure and blood vessel formation in natural aging and type-2 diabetes mice models

**Fig. 4:**
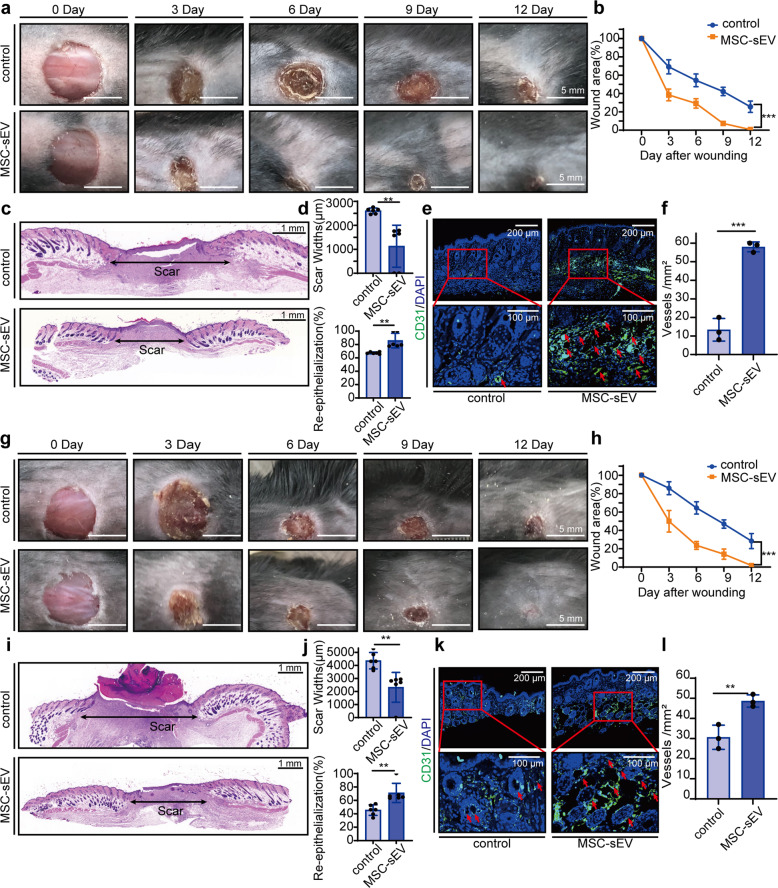
MSC-sEV promoted wound closure and blood vessel formation in natural aging and type-2 diabetes mice models. **a** Representative cutaneous wound photographs in senescent C57BL/6mice model at days 0, 3, 6, 9, and 12 after the operation treated with PBS (control), MSC-sEV 200 μg around wounds (scale bar, 5 mm). **b** Quantification of the wound areas during the wound-healing process. *n* = 6, ***p < 0.001 at Day 12. **c** H&E staining of the skin tissue around the wound at day 12 after the operation (horizontal arrows indicated the scar width; scale bar, 1 mm). **d** Quantification of the scar width and percentage of re-epithelialization around the wound. *n* = 6, **p < 0.01. **e** Representative immunofluorescence staining images positive cells of CD31 (green staining) and DAPI in paraffin-embedded sections of senescent C57BL/6mice dorsal skin injected PBS (control) or MSC-sEV. Red arrows indicate tubes formed around the wound (scale bar, 200 μm). **f** Quantification of vessels formed around the scar. *n* = 3, ***p < 0.001. **g** Representative dorsal wound photographs in type-2 diabetes C57BL/6mice model at day 0, 3, 6, 9, and 12 after the operation treated with PBS (control), MSC-sEV 200 μg around wounds (scale bar, 5 mm). **h** Quantification of the wound areas during the wound-healing process. *n* = 6, ***p < 0.001 at Day 12. **i** H&E staining of the skin tissue around the wound at day 12 after the operation (horizontal arrows indicated the scar width; scale bar, 1 mm). **j** Quantification of the scar width and percentage of re-epithelialization around the wound. *n* = 6, **p < *0.01*. **k** Representative immunofluorescence staining images positive cells of CD31 (green staining) and DAPI in paraffin-embedded sections of diabetic C57BL/6mice dorsal skin injected PBS (control) or MSC-sEV. Red arrows indicate tubes formed around the wound (scale bar, 200 μm). **l** Quantification of vessels formed around the scar. *n* = 3, **p < 0.01

The preventive effects of MSC-sEV on oxidative stress-induced senescence in vitro prompt us to investigate their role in wound-healing in vivo. To identify differences between normal and aged mice, we performed wound-healing surgery on 8-week-old mice and 64-week-old mice, and the results indicated that aged mice had a weaker ability of wound healing than younger mice (Supplementary Fig. 2a–c). And we subcutaneously injected PKH26-marked MSC-sEV to the mice dorsal skin, and found that MSC-sEV could be internalized by CD31-positive EC (Supplementary Fig. 2d). We used natural aging mice. Injection of 200 μg MSC-sEV significantly increased wound closure in aged mice as showed by smaller wound areas (Fig. 4a, b). H&E staining was carried out to evaluate the extent of re-epithelialization and scar formation. As shown in Fig. 4c, much wider newly formed epidermis and dermis with hair follicles and fat cells were observed in the wounds treated with MSC-sEV, as compared with the PBS-treated wounds at day 12. Quantitative measurements confirmed that MSC-sEV-treated wounds had a higher rate of re-epithelialization and a lower level of scar formation than the control group (Fig. 4d). Furthermore, MSC-exosomes-treated wounds exhibited significantly more newly formed blood vessels compared with the control wounds at day 12 post-wounding as demonstrated by immunostaining for endothelial marker CD31 and marker VE-cadherin (Fig. 4e, Supplementary Fig. 2e). Quantitative analysis of the density of new blood vessels confirmed that the beneficial effect of MSC-sEV on revascularization of the wounds (Fig. 4f, Supplementary Fig. 2f).

We further examined the functional role of MSC-sEV using a type 2 diabetes mouse model, in which EC senescence also play an important role in its pathogenesis^26^. Diabetic mice were established by injection of STZ and the digital photographs of wounds showed much faster-wound closure in diabetic mice treated with MSC-sEV (Fig. 4g, h). H&E staining showed the extent of re-epithelialization and scar formation in the wounds treated with MSC-sEV was much better than PBS-treated wounds at day 12 after operation (Fig. 4i). Quantitative measurements confirmed that MSC-sEV-treated wounds had a higher rate of re-epithelialization and a lower level of scar formation than the control group (Fig. 4j). Skin images from the undersurface revealed that MSC-sEV-treated wounds exhibited much more newly formed blood vessels compared with the control wounds at day 12 post-wounding (Fig. 4k, Supplementary Fig. 2g). Quantitative analysis of the density of new blood vessels confirmed that the beneficial effect of MSC-sEV on revascularization of the diabetic mice’s wounds (Fig. 4l, Supplementary Fig. 2h). Also, we performed immunofluorescence staining of SA-β gal and CD31 on aged mice and diabetes mice injected with PBS or MSC-sEV, the results showed that MSC-sEV decreased senescent EC number (Supplementary Fig. i–l). Taken together, our data suggested that MSC-sEV promoted skin recovery and angiogenesis in both natural aging and diabetic mouse models.

### MSC-sEV rescued senescent HUVEC through up-regulating miR-146a

**Fig. 5:**
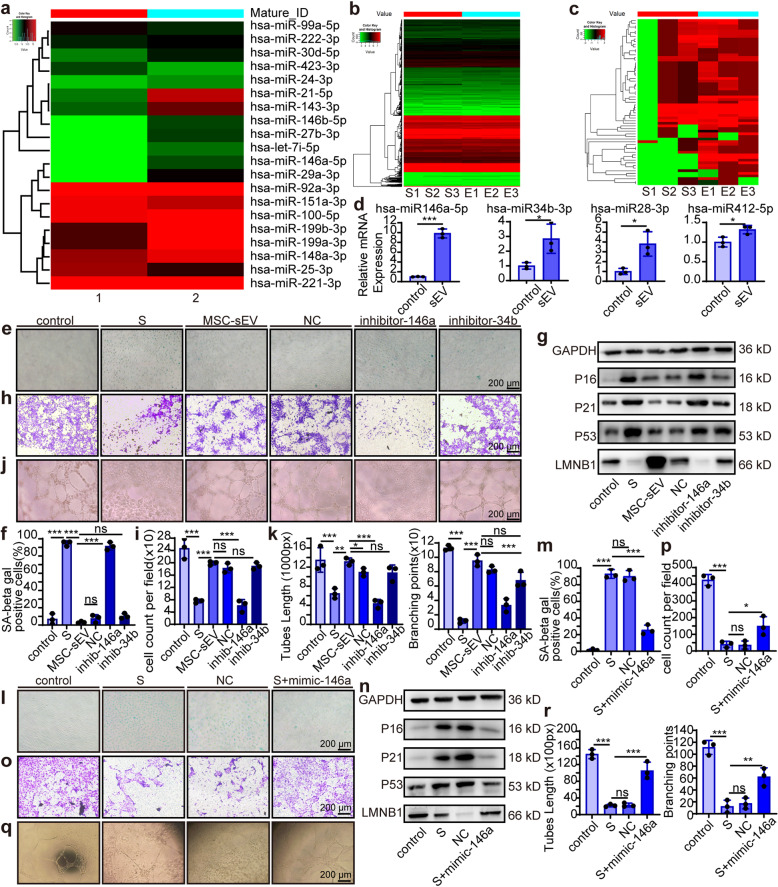
MSC-sEV rescued senescent HUVEC through up-regulating miR-146a. **a** The top 20 miRNAs in MSC-sEV were examined by Human miRNA Microarray, and the counts per million reads of miRNA in sample 1 and 2. The expression of the whole miRNome was analyzed in triplicate in senescent HUVEC (Sample S) and MSC-sEV-treated senescent HUVEC (Sample E). **b** Heat map showed all miRNAs upregulated in MSC-sEV-treated senescent HUVEC compared with senescent HUVEC. **c** Heat map showed the miRNAs that the upregulated fold change≥2 in MSC-sEV-treated senescent HUVEC compared with senescent HUVEC. (S represents senescent HUVEC, E represents MSC-sEV treated senescent HUVEC). **d** Quantitative of four of the upregulated miRNAs in HUVEC by qRT-PCR. *n* = 3, *p < 0.05, ***p < 0.001. HUVECs were treated with H_2_O_2_ before transfection, added MSC-sEV after transfection, and observed the changes after 24 h. (S represents senescent HUVEC; NC represents senescent HUVECs transfected with NC control RNA before treated with MSC-sEV in (**e**–**k**). **e** Representative images of SA β-gal staining (scale bar, 200 μm). **f** Quantitation of SA β-gal-positive cells in HUVEC. **g** Western blots analysis showing the change of senescence markers P16, P21, P53, and LMNB1 in HUVEC. **h** Representative images of transwell migration assays of HUVECs (scale bar, 200 μm). **i** Quantitation of transwell migration assays of HUVECs. *n* = 3, **p < 0.01, ***p < 0.001. **j** Representative images of in vitro angiogenesis assay (scale bar, 200 μm). **k** Quantitation of mean tube lengths and branching points in the HUVEC network. *n* = 3, **p < 0.01, ***p < 0.001. Senescent HUVECs were transfected with the mimics of miR-146a or with NC control RNA and observed the changes after 24 h. **l** Representative images of SA β-gal staining (scale bar, 200 μm). **m** Quantitation of SA β-gal-positive cells in HUVEC. *n* = 3, ***p < 0.001. **n** Western blots analysis showing the change of senescence markers P16, P21, P53, and LMNB1 in HUVEC. **o** Representative images of transwell migration assays of HUVECs. (scale bar, 200 μm). **p** Quantitation of transwell migration assays of HUVECs. *n* = 3, *p < 0.05, ***p < 0.001. **q** Representative images of in vitro tube formation assay (scale bar, 200 μm). **r** Quantitation of mean tube lengths and branching points in the HUVEC network. *n* = 3, *p < 0.05, **p < 0.01, ***p < 0.001

As a crucial component of exosome cargo, miRNAs have been reported to play important roles in mediating exosome functions. To identify which miRNA in MSC-sEV contributed to HUVECs senescence prevention, we first performed miRNA sequencing of MSC-sEV. Overall, we detected 1038 mature miRNAs, of which 382 had read counts over 100 and we listed the top 20 highly expressed in Fig. 5a. We then examined changes of miRNAs profile in senescent HUVECs after treatment with MSC-sEV and identified 875 upregulated and 645 downregulated miRNAs (Fig. 5b), among which 63 miRNAs with twofold increase after MSC-sEV treatment were selected (Fig. 5c). We postulated that MSCs exosomal miRNAs contributed to the upregulation of some of the 63 miRNAs, so we overlapped the top 200 miRNAs in MSC-sEV with the 63 miRNAs and found four miRNAs, hsa-miR-146a-5p, hsa-miR-34b-3p, hsa-miR-28-3p, and hsa-miR-412-5p. qPCR analysis confirmed that the four miRNAs were markedly increased in senescent HUVECs after exposure to MSC-sEV (Fig. 5d). We chose hsa-miR-146a-5p and has-miR-34b-3p for further analysis as these two miRNAs have been reported to be related to cell senescence. We postulated that if the effects of MSC-sEV were mediated through hsa-miR-146a-5p or has-miR-34b-3p, inhibition of their expression could abolish MSC-sEV effects. So, we transfected senescent HUVECs with respective inhibitors to reduce miR-146a (inhibitor-146a) and miR-34b (inhibitor-34b) before exposure to MSC-sEV. Our data showed that inhibitor-146a but not inhibitor-34b abolished MSC-sEV effects. After miR-146a inhibition, MSC-sEV could not reduce the percentage of SA-β gal positive cells (Fig. 5e, f), or downregulate p16, p21, p53, or upregulate LMNB1 expression levels (Fig. 5g, Supplement Fig. 3a). Moreover, after miR-146a inhibition, senescent HUVECs functions including migration and tube formation couldn’t be rescued by MSC-sEV (Fig. 5h–k). Whereas, inhibitor-34b had no effects (Fig. 5e–k). These results suggested that MSC-sEV effect was mediated through miR-146a, which prompted us to investigate whether miR-146a could exert preventive effect on senescent HUVEC. We increased miR-146a levels by using mimics of miR-146a (Supplementary Fig. 2b). As expected, mimic-146a decreased the percentage of SA b-gal positive cells (Fig. 5l, m), downregulated p16, p21, p53, and upregulated LMNB1 from both mRNA and protein level (Fig. 5n, Supplement Fig. 2c), increased migration and angiogenic capacity (Fig. 5o, p). Collectively, MSC-sEV rescued senescent HUVEC through up-regulating miR-146a.

### MSC-sEV rescued senescent HUVEC by downregulating Src signal pathway

**Fig. 6:**
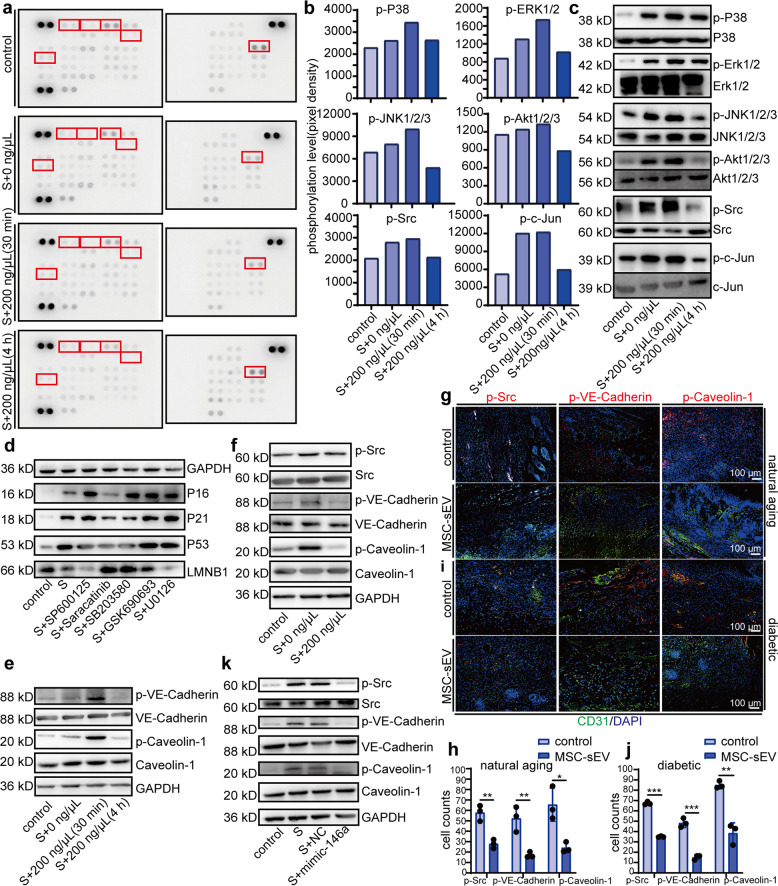
miR-146a rescue senescent HUVEC by downregulating Src signal pathway. **a** Phospho-kinase antibody array was performed on protein lysates from HUVEC pretreated with (S + 0 ng/μl MSC-sEV, S + 200 ng/μl MSC-sEV (30 min), S + 200 ng/μl MSC-sEV (4 H)) or without H_2_O_2_ (control). **b** Quantitation of different phosphorylation levels of P38, Akt1/2/3, Erk1/2, Src, JNK1/2/3, c-Jun which calculated from the array pixel density. The chosen protein was highlighted by red boxes in (**a**). **c** Western blot analysis showing phosphorylation of P38, AKT1/2/3, Erk1/2, Src, JNK1/2/3, c-Jun. **d** Western blots analysis showing the change of senescence markers P16, P21, P53, and LMNB1 in HUVEC treated with an inhibitor of JNK1/2/3(SP600125), Src (Saracatinib), P38 (SB203580), AKT1/2/3 (GSK690693), and Erk1/2 (U0126) before treated with H_2_O_2_ (50 μM, 2 h). **e** Western blots analysis showing the phosphorylation change of downstream of Src signal pathway VE-cadherin and Caveolin after co-cultured with MSC-sEV (200 ng/μl) for 30 min and 4 H compared with group control and group S (without MSC-sEV). **f** Western blots analysis showing the phosphorylation of Src, VE-cadherin, and Caveolin (downstream of Src) in high-glucose-induced senescent HUVEC. HUVEC was co-cultured with MSC-sEV (200 ng/μL, 48 h) after treated with high glucose (30 mM, 48 h). **g** Representative immunofluorescence staining images of positive cells of p-Src, p-VE-cadherin, p-Caveolin-1 (red staining), and CD31-positive cells (green staining) on paraffin-embedded sections of senescent C57BL/6mice dorsal skin injected PBS (control) or MSC-sEV (scale bar, 100 μm). **h** Quantitation of positive cells of p-Src, p-VE-cadherin, p-Caveolin-1 in CD31-positive cells. **i** Representative immunofluorescence staining images of positive cells of p-Src, p-VE-cadherin, p-Caveolin-1 (red staining), and CD31-positive cells (green staining) on paraffin-embedded sections of type-2 diabetes model C57BL/6mice dorsal skin injected PBS (control) or MSC-sEV (scale bar, 200 μm). **j** Quantitation of positive cells of p-Src, p-VE-cadherin, p-Caveolin-1 in CD31-positive cells. **k** Western blots analysis showing the phosphorylation change of Src, VE-cadherin, and Caveolin (downstream of Src) after transfected mimic-146a in HUVEC

To identify which signaling pathways were affected by MSC-sEV, we performed phospho-kinase antibody array in normal HUVECs, senescent HUVECs and senescent HUVECs treated with MSC-sEV for 30 min and 4 h. As showed in Fig. 6a, of the 43 kinases examined, 12 was detected to have an increase of phosphorylation in senescent HUVECs. After exposure to MSC-sEV for 4 h, 8 of the 12 phosphorylated kinases were recovered (Fig. 6b). Western blot confirmed the change of the phosphorylation status during the progress (Fig. 6c). All these results suggested that MSC-sEV could inhibit kinases phosphorylation. We then investigated whether inhibition of these kinases could recapitulate MSC-sEV effects. Senescent HUVECs were treated with inhibitor of JNK1/2/3 (SP600125), Src (Saracatinib), P38 (SB203580), AKT1/2/3 (GSK690693), and Erk1/2 (U0126), respectively, and western blot indicated that inhibition of Src suppressed HUVECs senescence (Fig. 6d). We also found that inhibition of Src could improve senescent HUVECs functions, including migration and tube formation (Supplementary Fig. 4a–e). Western blot showed that the downstream of Src, VE-cadherin^27^ and Caveolin-1^28^ were activated in senescent HUVECs and recovered by MSC-sEV (Fig. 6e). Additionally, we found MSC-sEV recovered phosphorylation of Src, VE-cadherin and Caveolin-1 in high-glucose-induced senescent HUVECs, the same as in H_2_O_2_-induced senescent HUVECs (Fig. 6f). Furthermore, immunofluorescence staining showed that MSC-sEV-treated wounds exhibited much less phosphorylated Src, VE-cadherin and Caveolin-1 in CD31-positive cells in both natural aging and diabetic mice wound-healing models, (Fig. 6g–j). To investigate whether miR-146a could reduce the phosphorylation of Src, VE-cadherin and Caveolin-1 in senescent HUVECs, we used mimics of miR-146a to treat senescent HUVECs. Western blot showed that mimic-146a reduced phosphorylated Src, VE-cadherin and Caveolin-1 in senescent HUVECs (Fig. 6k). In summary, our findings suggest that MSCs exosomes rescued senescent HUVEC by downregulating Src signal pathway and this effect was mediated through miR-146a.

## Discussion

**Fig. 7:**
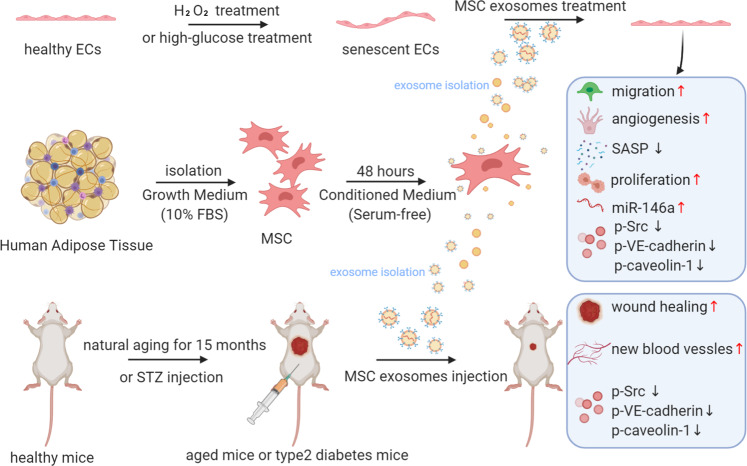
Schematic illustrations of MSC-sEV repairments on oxidative stress-induced senescence ECs in vitro and in vivo via regulation of the miR-146a/Src pathway (Created with BioRender.com)

This study was aimed to investigate the therapeutic effects and mechanism of MSC-sEV on oxidative stress-induced HUVECs senescence. We found that in senescent HUVECs, the MSC-sEV treatment protected against senescence-induced biomarkers and dysfunctions, improved angiogenesis, migration, and proliferation capacities, SASP, mitochondrial dysfunction, and ROS level. Furthermore, our data indicated that MSC-sEV could promote skin wound healing in aging and type-2 diabetes mice model after subcutaneous injection of MSC-sEV, suggesting that MSC-sEV may be a potential nanotherapeutic agent for the treatment of vascular-related diseases or disorders, the pathological basis of which involves senescence (Fig. 7).

The use of the traits, included cell function alternations, morphology, gene expression, and positive staining for SA β-gal in combination is the current best practice for identifying senescent cells^5^. Thus, we established a convictive HUVECs senescence model and found out the concentration of MSC-sEV to rescue senescent HUVECs efficiently in vitro through comprehensive traits mentioned above in this research. EVs derived from stem cells play a key role in mediating the capacity of stem cells through the delivery of biologically active molecules into recipient cells^19^. Several studies have shown the effects of stem cell-derived EVs in the disease therapy field^16,21,23,29^. Antonio Casado-Díaz et al. demonstrated that MSC-derived EV is beneficial to the chronic skin ulcers treatment, including decreasing scar formation and accelerating healing^16^. MSC-sEV can be in the treatment of neurological diseases such as epilepsy, stroke, or traumatic brain injury which derived from strong anti-inflammatory effects^20^. Adipose MSC sEV through Wnt/β‐catenin signaling improve cell proliferation, migration, and restrain cell apoptosis in skin wound healing^23^. However, no study reported the effects and underlying mechanisms of MSC-sEV action with respect to ECs senescence. In this research, we first extensively tested some important functions to estimate whether MSC-sEV promote the functional recovery of senescent ECs following identifying senescent biomarkers in HUVECs. EC senescence can impair the repair capacity of the endothelial lining as a direct result of permanent growth arrest^30^. An in vitro migration and tube formation assay showed that MSC-sEV could improve the functional recovery of senescent HUVECs compared with the group without sEV. Although the cell proliferation capacity enhanced by MSC-sEV may affect the rate of gap-filling of the scratch wound, the effect of HUVECs on the migration of HUVECs was demonstrated in the transwell migration assay. These results indicate that MSC-sEV contain key factors to restore the proliferation and migration capacity of senescent HUVECs. As the senescence-associated secretory phenotype (SASP), for what is known, senescent cells generate proinflammatory and matrix-degrading molecules, and the SASP discovery proposed a mechanism by which senescent cells could influence tissue and organ function out of proportion to their numbers^31^. Eliminating senescent cells and attenuating the SASP have emerged as promising therapeutic strategies due to their possible role in many aging and disease processes^5^. ELISA tests showed in Fig. 3g suggested that MSC-sEV reduced the increase in SASP induced by senescence (IL-1alpha, IL-6, and IL-8). Substantial evidence shows that reactive oxygen species (ROS) generated from either intracellular or extracellular sources in ECs can induce or accelerate the development of senescence by acting at multiple subcellular levels^7^. Our results showed that MSC-sEV treatment in the culture of senescent HUVECs can prevent the formation of oxidative stress-induced ROS and DNA damage (Fig. 3h). Endothelial mitochondria are recognized to play a prominent role in signaling cellular responses to environmental signals^6^. Mitochondria are not the key source of ATP in ECs, but acting as ROS signaling organelles, preserving EC homeostasis^32^. Our experiments demonstrated that HUVECs mitochondria dysfunction induced by senescence can be ameliorated by MSC-sEV treatment. Together, these results suggested that MSC-sEV rescue ECs dysfunction caused by senescence comprehensively.

Senescence of ECs can cause atherosclerosis as a prevalent component of cardiovascular diseases^6^. In the pathogenesis of heart failure, endothelial senescence has been implicated^33^. It is also known that premature EC senescence plays a key role in the pathogenesis of diabetic vascular problems, including diabetic retinopathy^10^. Angiogenesis usually forms new vessels to help with wound healing in many progresses^34^. Angiogenesis alterations are associated with age, since the wound-healing process is always delayed in older people compared to younger people^2^. Diabetics leads to delayed wound healing by affecting angiogenesis^12^. In our experimental setting, aging and STZ is used as a source of endogenous oxidative stress in vivo, which is consistent with H_2_O_2_ and high-glucose-induced HUVEC senescence in vitro. We used natural aged mice to investigate natural ECs senescence, and STZ-injection caused a diabetes model with extremely high levels of circulating glucose that enabled vascular senescence to be induced in a short period of time. In this research, we did not conduct an in-depth investigation into the impact of MSC-sEV on cardiac function, but EC senescence and diabetes led to less angiogenesis and wound healing, which could be rescued by MSC-sEV. Young-Mee Kim et al. showed that defective wound healing and angiogenesis in type 2 diabetes mice caused by EC senescence were rescued by EC-targeted PDIA1 or mutant Drp1^32^. Qianfa Long et al. identified that MSC-sEV intracutaneous injection reduced UV-induced histological injury and inflammatory reaction in mice skin.^35^. Hui Xie et al. demonstrated that human urine-derived stem cells promoted wound healing in streptozotocin-induced diabetic mice by promoting angiogenesis via transferring DMBT1 protein^36^. Our research is the primary, to our knowledge, to show that local transplantation of adipose-derived-MSC-sEV into full-thickness excisional skin wounds was able to induce significant regenerative effects through recovering angiogenesis of senescent ECs in aged and diabetic mice, as characterized by faster wound closure, higher rates of re-epithelialization and EC proliferation, as well as more vessel formation. Here, we observed that the amount of newly developed blood vessels in diabetic wounds was substantially enhanced by MSC-sEV, which indicated that MSC-sEV-induced pre-wound-healing action is likely to be attributable to their stimulatory effects on senescent EC recovery. Every year, poor wound healing after trauma, surgery, acute disease, or chronic disease conditions affects millions of people worldwide and is the result of poorly regulated healthy tissue repair response elements, including inflammation, angiogenesis, deposition of matrices, and recruitment of cells; in general, failure of one or more of these cellular processes is related to an underlying pathological disorder such as vascular disease, diabetes, or aging, both of which are often linked to pathologies of healing^37^. Our wound-healing research might enhance the natural repair mechanisms of the body for clinical strategies.

miRNAs from exosomes which derived from stem cells play a key role in mediating the capacity of stem cells through the delivery into recipient cells^19^. It is well recognized that miRNAs, which perform biological functions by controlling RNA targets post-transcriptionally, are a class of non-coding single-stranded RNAs^38^. Through investigation, we found that to recover senescent ECs, miRNAs had a significant influence on the function of MSC-sEV. We found four miRNAs, hsa-miR-146a-5p, hsa-miR-34b-3p, hsa-miR-28-3p, and hsa-miR-412-5p by overlapping miRNA microarray results that may rescue the senescent ECs. After literature review, we found that miR-146a and miR-34b may involve in senescence progress in types of cells^39–42^. We used inhibitors of miRNAs to identify the effect on HUVECs senescence, and found that inhibition of miR-146a aggravated the HUVECs senescence and dysfunction but not miR-34b. And then we identified that the mimics of miR-146a could rescue senescent HUVECs. miR-146a deficiency has been reported to worsen whether in cardiac function, cardiomyocyte apoptosis or baseline autophagy^41^, and involved in inflammation activation in high-fat treated endothelium by directly regulating IRAK1 and TRAF6^39^, and also related to HG-induced endothelial inflammation control through IRAK-1 modulation^40^. Moreover, miR-146a-5p partly dedicated to the treatment of group 2 innate lymphoid cell-dominant allergic airway inflammation^43^. Rau CS et al. discovered miR-146a may play a role in regulating the angiogenesis in HUVECs by downregulating CARD10, which acts in a negative feedback regulation loop to inhibit the activation of NF-κB that normally impairs angiogenesis^44^. Su ZF et al showed that miR-146a/b promoted proliferation, migration and angiogenesis ability of endothelial progenitor cells through downregulation of TRAF6 and IRAK1 expressions^45^. And Li Y et al also found that miR-146a-mediated TGF inhibition, contributing to angiogenesis. However, most of these studies focused on the effect of cellular miR-146a^46^. However, to our knowledge, no study has shown a link between MSC-sEV and miR-146a in the regulation of EC senescence. Altogether, our research suggests that miR-146a-5p may be a novel target for the treatment of cardiovascular diseases and diabetic vascular complications via rescue senescent ECs. We then investigated which protein influenced the senescent ECs through MSC-sEV. We found that among JNK1/2/3, Src, P38, AKT1/2/3, and Erk1/2 which we selected to increase the senescence of HUVECs, inhibition of Src decreased HUVECs senescence the most. The Src kinase family has been implicated in aging processes, Judith Haendeler et al. showed that the activity of the Src kinase family enhanced by ROS accumulation with age and induce lipid peroxidation, protein modification, DNA strand breaks, and oxidative damage, which results in a progressive loss of cell function, a hallmark for aging processes, and suggested that H_2_O_2_ induced ECs senescence was completely blocked with the Src-family kinase inhibitor PP2^47^, which was consistent with our results. Sascha Jakob et al. also showed that Src could play a role in aging processes, since Src is responsible for TERT tyrosine phosphorylation, and Src deficiency can retain telomerase reverse transcriptase (TERT) in the nucleus under oxidative stress. We provided functional evidence that Src, the downstream of Src, VE-cadherin and Caveolin-1, were phosphorylated by senescence, and MSC exosomes inhibited the activation of Src both in H_2_O_2_ induced senescent HUVECs and high-glucose induced senescent HUVECs. This phenomenon had been identified in aged and type-2 diabetes C57BL/6mice model. We also found that mimic-146a could inhibit the activation of Src kinase, which meant that MSC-sEV regulated the activation of Src partially through miR-146a. To find out how miR-146a regulate SRC, we first searched for the targets of miR-146a through miRTarBase (http://miRTarBase.cuhk.edu.cn/)^48^, and we found 11 experimentally validated targets. However, Src is not among these validated targets, suggesting that miR-146a doesn’t regulate Src directly. We postulated that one or more molecules might be involved in the regulation between miR-146a and Src. Further experimental data are needed, and we will continue this investigation in the future.

In short, our findings show that MSC-sEV can effectively increase senescent EC recovery and accelerate the healing of aged and diabetic mice wounds through increased angiogenesis. In the process of regulating functional recovery and wound healing dependent on MSC-sEV, miR-146a and Src play a crucial role, miR-146a can partially promote the pro-angiogenic and wound-healing effects, and subsequently dephosphorylate Src. Our findings suggest that by recovering senescent ECs via miR-146a, MSC-sEV may represent a promising strategy for vascular aging induced disease.

## Materials and methods

### Cell culture

Human adipose tissues and umbilical cords were obtained according to procedures approved by the Ethics Committee at Peking Union Medical College and the Chinese Academy of Medical Sciences. MSCs were isolated from human adipose tissues of healthy volunteers and culture-expanded as previously reported^29^. HUVECs were isolated from human umbilical cords and cultured in endothelial cell medium (ECM #1001; ScienCell), following protocols described before^49^. MSCs of passage 5 and HUVECs of passage 3 or 4 were used for in our experiments. Cells were cultured in a humidified incubator with 5% CO_2_ at 37 °C and passaged with trypsin/EDTA after reaching the confluence.

### Transmission electron microscopy

The morphology of MSC-sEV was examined by transmission electron microscope. On a carbon-coated copper grid, 20 μL of exosome suspension was simply loaded and set for at least 5 min. The MSC-sEV were subsequently stained with 2% uranyl acetate and dried for 10 min. Then, the grids were visualized with transmission electron microscope (Tecnai G2 Spirit TEM, Zeiss, Oberkochen, Germany) at 120 kV.

### Nanoparticle tracking analysis

Nanoparticle Tracking Analysis (Zetaview, Particle Metrix) was used to evaluate the size distribution of MSC-sEV. Based on Brownian motion and diffusion coefficient, the particles are tracked and sized automatically. MSC-sEV is diluted to 1.0 mL with PBS. The measurement conditions for each sample were 23.75 ± 0.5 °C, 25 frames per second (FPS) for 60 s, with a similar detection threshold.

### Exosomes internalization assay

MSC-sEV were labeled with a red fluorescent dye (PKH26; Sigma) according to the manufacturer’s instructions. The labeled sEV were then added to HUVECs or senescent HUVECs and co-cultured for 30 min, 2, 4, and 6 h. HUVECs were washed with PBS and fixed in 4 % paraformaldehyde for 15 min. Nuclei were stained with Hoechst, and the signals were analyzed with a fluorescence microscope.

To investigate mechanisms for internalization of MSC-sEV by HUVEC, we applied various endocytic inhibitors. Chlorpromazine (CPZ) is a widely used chemical inhibitor to interact with clathrin-mediated endocytic route (CME) by preventing clathrin lattices from being installed on endosomal membranes and clathrin-coated pits from being formed on the cell surface^50^. Nystatin is known to interfere with the caveolin-1 related uptake, which is associated with caveolae formation^51^. 5-(N-ethyl-N-isopropyl) amiloride (EIPA) is an anti-inhibitor of Na+/H+, thus inhibiting endocytosis mediated by macropinocytosis^52^. We used CPZ (20 μM), nystatin (50 μM), and EIPA (50 μM) treated HUVECs for 30 min before the PKH26-labeled MSC-sEV were added.

### Isolation of sEV from MSC

We followed the MISEV 2018 guidelines to isolate and identify MSC-sEV^53,54^. Briefly, after MSCs reached 80–90% confluence, the serum-free medium was added for 48 h to avoid contamination of vesicles from serum. The conditioned medium was collected and centrifuged 800 g for 5 min and additional 3000 g for 10 min to remove cells and debris. The supernatant was then subjected to a 0.1-mm-pore polyetherrsulfone membrane filter (Corning) filtration to eliminate cell debris and large vesicles, followed by a 100,000-Mw cutoff membrane concentration (CentriPlus-70, Millipore). The supernatant volume was reduced to less than 5 mL from approximately 250–500 mL. Using the 70Ti Rotor, the supernatant was then ultra-centrifuged at 110,000 *g* for 2 h at 4 °C (Beckman Coulter). The resulting pellets were resuspended with PBS and ultra-centrifuged with 100 Ti Rotor for 1 h at 110,000 *g* at 4 °C (Beckman Coulter). We used phosphate-buffered saline (PBS) as a negative control in the experiments involving MSC-sEV.

### Induction of HUVECs senescence

H_2_O_2_ and high glucose were used to induce HUVEC senescence. Cells at 70% confluence were exposed to different doses of H_2_O_2_ (0, 25, 50, 75, and 100 μM) for 2 h and then washed with PBS and changed to fresh media. To get high-glucose-induced senescent HUVECs, we treated HUVECs with 30 mM d-glucose (HG) for 48 h^9^. Control group was cultured in the media with normal glucose alone (5.5 mM).

### Quantitative real-time polymerase chain reaction(qRT-PCR)

TRIzol was used to extract the total RNA. At 260 and 280 nm, RNA concentrations and purity were calculated by optical density. Real-time PCR amplification was carried out in triplicate. SYBR Green RT-PCR (Takara Biotechnology Co., Ltd., Tokyo, Japan) was used to cDNA fragments for quantitative PCR. Relative mRNA expression was assessed using the 2-ΔΔCt method and normalized to GAPDH expression.

### Western Blotting

Radioimmunoprecipitation (RIPA) lysis buffer with PMSF extracted proteins and the BCA Protein Assay Kit were used to quantify proteins. We performed Western Blot in triplicates followed the procedures reported previously^55^ and used GAPDH as the internal control.

### Immunofluorescence staining

Cultured cells were fixed for 10 min at 4 °C in ice-cold methanol, washed three times in phosphate-buffered saline (PBS) and then at room temperature permeabilized in 0.1 percent Triton X-100/PBS for 10 min. With 0.5 percent Tween-20/PBS containing 1 percent bovine serum albumin (BSA) for 30 min, nonspecific binding was blocked. At 4 °C overnight, the primary antibodies were incubated. We used antibodies VWF (1:500; rabbit IgG) and CD31(1:500; rabbit IgG) for experiments. The secondary antibodies were, at room temperature, incubated for 1 h. PBS washed the incubated cells, and Hoechst was used to label the nuclei.

### Transwell assay

We performed transwell assays by using 24-well transwell inserts with 8 μm pore-sized filters and 24-well culture plates (Corning, NY, USA). Cells were suspended in a low serum medium (containing 3% FBS) and plated into the upper chamber (1 × 10^5^ cells per well; three replicates per group). In the lower chamber, 500 μL of complete medium (containing 10% FBS) were added. Cells attached to the upper surface of the filter membranes were removed using cotton swabs after 12 h of incubation and cells on the lower side of the filter (the migrated cells) were stained with crystal violet.

### Scratch wound-healing assay

Cells (2 × 10^5^ cells per well; three replicates per group) were seeded and incubated at 37 °C in a 12-well plate. We scratched the monolayer with a p200 pipette tip after the cells had been attached, washed with PBS to remove floating cells. At 0 h, 6 h and 12 h later, HUVECs were photographed. The migration area rate was determined as the closure area to initial wound ratio. Migration area (%) = (A1 – A0)/A0 × 100, where A1 represents the width of initial wound area and A0 represents the remaining width of wound at the metering point which analyzed by Image J software.

### Tube formation assay in Matrigel

By conducting a tube formation assay, in vitro capillary network formation was determined in Matrigel. 1 × 10^5^ HUVECs were plated in triplicates with 100 uL serum-free medium on a growth-factor-reduced Matrigel (BD)-coated 96-well plate. Tube formation was examined by microscopy after 6 h of incubation (Olympus, Tokyo, Japan) and branch points and tube length were quantified by choosing three fields per well at random with Image J software.

### Enzyme linked immunosorbent assay (ELISA)

HUVECs conditioned medium was collected from each group (*n* = 3 per group) after PBS or MSC-sEV co-cultured for 24 h, stored at room temperature for 15 min and then centrifugated at 2000 g for 10 min. The samples were either frozen at −80 °C or immediately analyzed using human ELISA kits for IL-1alpha, IL-6, and IL-8. We performed all procedures by following the manufacturer’s instructions and measured the absorbance at 450 nm by using a microplate reader (Bio-Rad, USA). Each determination was the average of at least three independent experiments.

### Mitochondrial respiration analysis using the Seahorse XF Analyzer

To calculate the oxygen consumption rate (OCR), the Mito Stress Test Kit was used. The probe plate was hydrated with HPLC grade water in a CO_2_-free incubator prior to metabolism calculation. In order to preserve the pH value, the test phenol red-free solution containing 10 mM glucose, 2 mM glutamine, 1 mM pyruvate and 5 mM HEPES was kept in a 37 °C CO_2_-free incubator. In the hydration plate, the HPLC grade water was then replaced with a calibration solution and stored in a CO_2_-free 37 °C incubator. When density of cells was at 5000 per well, HUVECs were seeded into XF96 cell culture microplates (Seahorse Bioscience) overnight and allowed to adherent to the tray. Then the cell culture medium was substituted with a red-free phenol assay solution and put for 1 h in a 37 °C CO_2_-free incubator. Finally, the mitochondrial oxidative phosphorylation and glycolysis output rates of OCR were calculated and analyzed according to the manufacturer’s instructions and protocols on the Agilent Seahorse Bioscience XF96 Extracellular Flux Analyzer (Agilent Technologies) (Seahorse Bioscience, North Billerica, MA, USA).

### Reactive oxygen species (ROS) detection

Intracellular ROS generation in HUVECs was measured by using Fluorometric Intracellular ROS Kit. The HUVECs of each group (*n* = 3 per group) were seeded onto 6-well plates at the density of 3 × 10^5^ cells per well in the culture medium. After the cells reached 80 % confluence, they were subjected to H_2_O_2_ stimulation, and then incubated with MSC exosomes for 12 h. After the indicated time, the cells were incubated with ROS Kit for 30 min at 37 °C. Finally, these cells were washed with pre-warmed PBS and tested using on a flow cytometer (BD, Accuri C6 Cytometer, USA) within 1 h. We performed each experiment at least for 3 samples and each determination was made in triplicate.

### Proliferation assay

We seeded cells into 96-well culture plates for 5 × 10^3^ cells per well and each group had four replicates. A no-cell community acted as the blank one. MTS reagents were applied to the culture medium on days 0, 1, 2, 3, and 4 (100 μL per well). We measured the absorbance at 490 nm in each well by a microplate reader after incubation for 1 h at 37 °C (Bio-Rad 680, Hercules, USA) and cell proliferation was represented by each individual well’s mean absorbance minus the blank value of each well.

### Mouse skin wound model and treatments

We purchased aged female C57BL/6 J mice normal male C57BL/6 J from the Laboratory Animal Center of Peking Union Medical School (Beijing, China). The mice were 64-week old when we performed surgery, and the weight of the mice was 35–40 g. Male C57BL/6 J mice of 8-week old were used and type 2 diabetes mouse model was established as reported^56^. Briefly, for the first 12 weeks, 16 mice were fed a high-fat diet (HFD). Following that, the HFD mice were given an intraperitoneal injection of STZ for 7 days (30 mg/kg). STZ was dissolved with Citrate–phosphate buffer which was a mixture of citrate and phosphate and the solution of STZ was freshly prepared every day before injection. Blood glucose levels were checked one week following the final injection, and mice with random blood glucose levels more than 16.7 mmol/l were classified as type 2 diabetic mice. We followed animal use and experimental procedures approved by the Animal Care and Use Committee of Peking Union Medical School. The mice were held for one week under observation prior to experimental procedures. The mice were anesthetized with a 50 mg/kg pentobarbital sodium (Sigma-Aldrich) intraperitoneal injection and a full-thickness excisional skin wound (8 mm diameter) was made after shaving on the back of each mouse. The mice were separated into two groups at random (n = 6): PBS group (treated with 100 μL PBS), MSC-sEV group (treated with 200 μg MSC-sEV in 100 μL PBS). Briefly, they were subcutaneously injected with MSC-sEV or PBS around the wounds at 4 injection sites (25 μL per site). When on days of 0, 3, 6, 9, and 12 post-wounding, wounds were photographed and measured. At 12 days post operation, mice were sacrificed, and skin samples were harvested and fixed in 10% paraformaldehyde (PFA). Samples from each group were subjected to hematoxylineosin (H&E) staining for the detection of newly formed blood vessels.

### Matrigel plug assay

Growth-factor-reduced Matrigel aliquots (150 μL) containing 5 × 10^6^ HUVECs pretreated with H_2_O_2_ or MSC-sEV were prepared on ice. Mice were randomly divided into three classes (control, S and MSC-sEV, *n* = 3) and anaesthetized with a 50 mg/kg pentobarbital sodium intraperitoneal injection (Sigma Aldrich). Matrigel aliquots were injected into the inguinal areas bilaterally and could gel at body temperature. The mice were euthanized for plug excision after 14 days. Plugs were fixed with 4 % PFA and stained with anti-human CD31 for angiogenesis detection.

### Transfection of mimic and inhibitor

HUVECs in 6-well plates (about 5 × 10^5^ cells/well) were transfected with miR-146 mimic (50 nmol/L), or miR-146 inhibitor, or miR-34 inhibitor (100 nmol/L), or their corresponding negative controls. Lipo2000 transfection reagent was simultaneously added into the medium for efficient transfection. After 6 h, we replaced the culture medium in order to remove the transfection reagent. Detection was made 24 h after transfection. miR-146a mimic, miR-146a inhibitor, miR-34b inhibitor, and their negative controls were purchased from Sangon Biotech.

### Oil red O staining, Alizarin red staining and Alcian Blue staining

After lineage differentiation, cells were washed twice with PBS and fixed with 4 % formalin for 10 min at room temperature. For Oil red O staining, fixed cells were then stained with Oil red O reagent (Beyotime) for 1 h at room temperature; for Alizarin red staining, fixed cells were stained with 1% Alizarin red (Leagene, Beijing, China) with pH 4.2 for 30 min at room temperature; for Alcian Blue staining, fixed cells were then colored for 30 min in a 1% Alcian Blue solution (OriCell). Finally, cells were washed with water for three times to remove unbound dye and photographed under light microscopy.

### Flow cytometry analysis

MSC immunophenotype analysis was performed as previously reported^29^. Briefly, MSCs were washed and incubated with primary antibodies (CD29, CD34, CD44, CD45, CD73, CD90, CD105, CD106, CD206, and HLA-DR, BD Biosciences) for 30 min at 4 °C. After washing, secondary antibodies were used to incubated with MSCs for 30 min at 4 °C, and MSCs were analyzed by flow cytometer and CFlow Plus software.

### Statistical analysis

We presented mean ± SD for all data. We analyzed the comparisons between groups using Student’s t test, one-way ANOVA followed by Tukey’s multiple comparisons test and Dunnett’s multiple comparisons test. Statistically significant differences were considered at *p < 0.05, **p < 0.01, ***p < 0.001.

## Supplementary information

Supplementary Materials

## Data Availability

All data supporting this paper are present within the paper and/or the Supplementary Materials. The original datasets are also available from the corresponding author upon request.
