# Correction to: Mesenchymal stem cell-derived small extracellular vesicles mitigate oxidative stress-induced senescence in endothelial cells via regulation of miR-146a/Src

**DOI:** 10.1038/s41392-022-01075-y

**Published:** 2022-07-14

**Authors:** Xian Xiao, Meiqian Xu, Hongliang Yu, Liping Wang, Xiaoxia Li, Janusz Rak, Shihua Wang, Robert Chunhua Zhao

**Affiliations:** 1 Institute of Basic Medical Sciences Chinese Academy of Medical Sciences, School of Basic Medicine Peking Union Medical College, Beijing, China; 2 Department of Genetics and Cell Biology, Basic medical college, Qingdao University, 308 Ningxia Road, 266071 Qingdao, China; 3 Research Institute of the McGill University Health Centre, Glen Site, McGill University, Montreal, QC H4A 3J1 Canada; 4 Department of Cell Biology, School of Life Sciences, Shanghai University, 200444 Shanghai, China

**Keywords:** Senescence, Mesenchymal stem cells

Correction to: *Signal Transduction and Targeted Therapy* 10.1038/s41392-021-00765-3, published online 22 October 2021

In the process of collating the raw data, the authors noticed several inadvertent mistakes occurred in Fig. 1b, Fig. 2d, f, l, and Supplementary Fig. 1b that need to be corrected after online publication of the article^1^. The correct data are provided as follows. The key findings of the article are not affected by these corrections. The original article has been corrected.

1. Scale bar in Fig. 1b was mislabeled as 100 μm, which should be 100 nm.

**Fig. 1:**
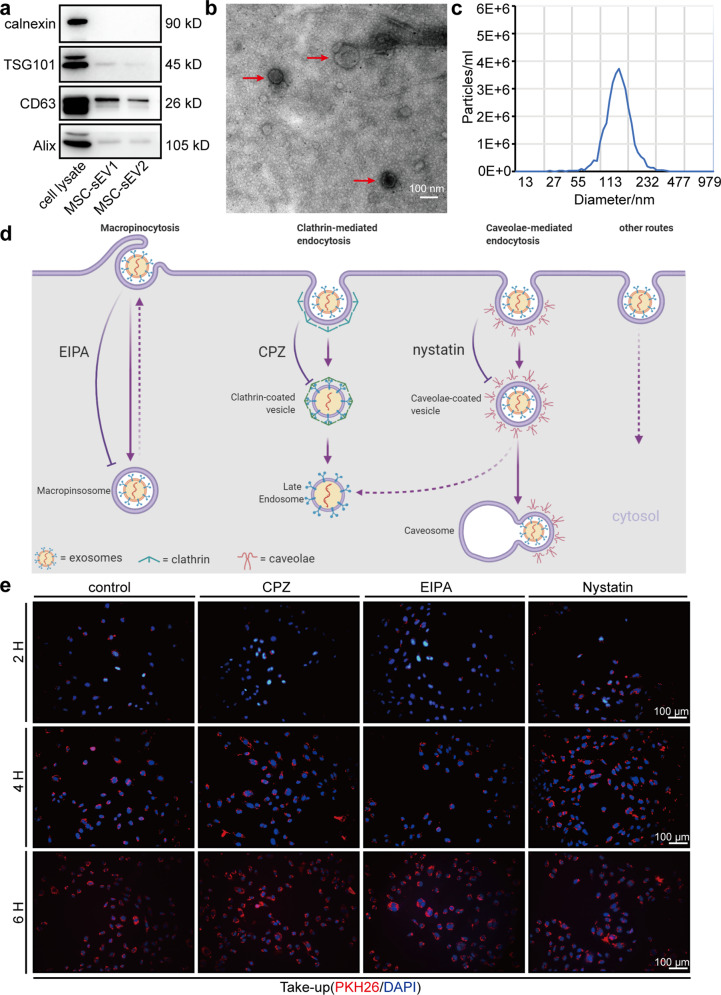
**b** Transmission electron microscopic images of MSC-sEV (scale bar, 100 nm).
2. During the preparation of Fig. 2, the representative image showing P21 expression in Fig. 2d, SA β-gal staining of S+100 ng/μL in Fig. 2f, and GAPDH expression in Fig. 2l were pasted and placed by mistake. The correct results should be as shown below.

**Fig. 2:**
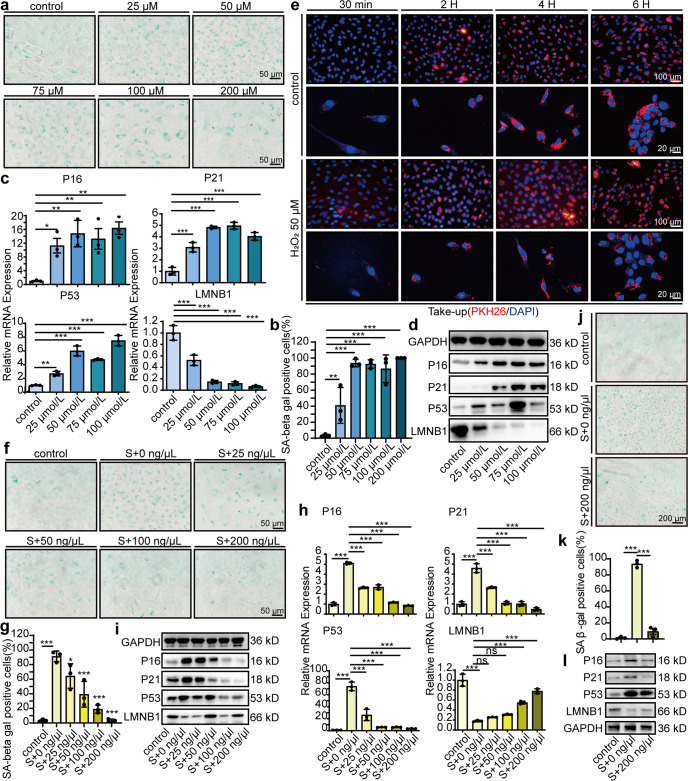
**d** Representative images of western blot analysis showing the senescence markers P16, P21, P53, and LMNB1 in HUVEC after 48 hours of 2 h pre-treatment with different concentrations of H_2_O_2_ (25 μmol/L, 50 μmol/L, 75 μmol/L, 100 μmol/L). **f**. Representative images of SA β-gal staining in HUVECs (scale bar, 50 μm). HUVECs were incubated with 0 ng/μL, 25 ng/μL, 50 ng/μL, 100 ng/μL and 200 ng/μL MSC-sEV for 48 h after pretreated with H_2_O_2_ (50 μmol/L, 2 h). **l**. Representative images of western blot analysis showing the changes of senescence markers P16, P21, P53 and LMNB1 in high-glucose-induced senescent HUVECs. HUVECs were cultured in the media with 30 mM d-glucose for 48 h to induce senescence and then incubated with PBS (S+0 ng/μL) or 200 ng/μL MSC-sEV (S+200 ng/μL) for 48 h. HUVECs cultured in the media with normal glucose 5.5 mM were used as control.
3. During the preparation of Supplementary Fig. 1b, images of CD29, CD44 and CD105 were distorted by mistake. The correct results should be as shown below.

**Figure:**
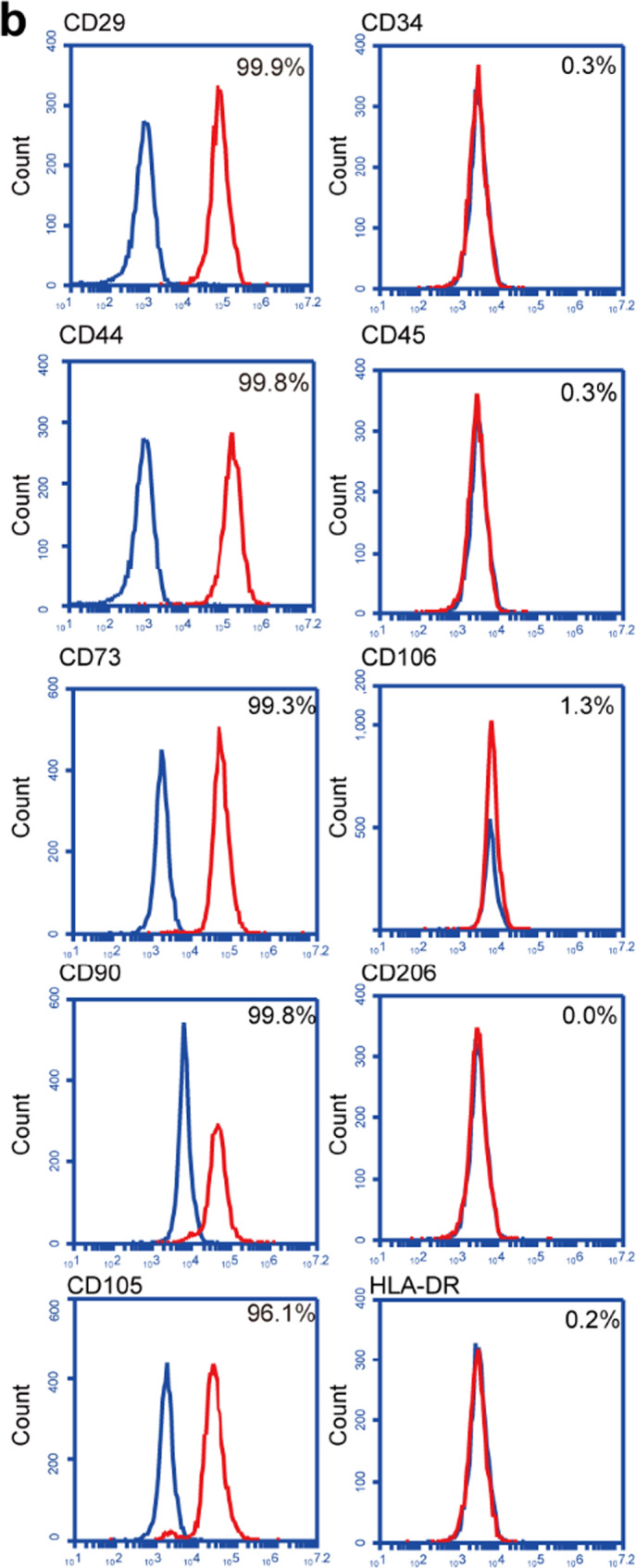
Fig. S1b Flow cytometry analysis found MSC markers CD29, CD44, CD73, CD90, CD105 are positive, and CD34, CD45, CD106, CD206, HLA-DR are negative.

## Supplementary information

Supplementary Materials

Revised Supplementary figure 1

## References

[1] Xiao X (2021). Mesenchymal stem cell-derived small extracellular vesicles mitigate oxidative stress-induced senescence in endothelial cells via regulation of miR-146a/Src. Sig. Transduct. Target. Ther..

