# Supplementary material for: Mesenchymal stem cell-derived small extracellular vesicles mitigate oxidative stress-induced senescence in endothelial cells via regulation of miR-146a/Src
Source: Signal Transduct Target Ther. 2021 Oct 22;6:354. doi: 10.1038/s41392-021-00765-3 (PMC8531331; doi:10.1038/s41392-021-00765-3)
Supplement: Supplementary file 1 — Supplementary Materials [file 41392_2021_765_MOESM1_ESM.docx]

Supplementary Materials for

**Mesenchymal stem cell derived small extracellular vesicles mitigate oxidative stress-induced senescence in endothelial cells via regulation of miR-146a/Src**

Xian Xiao^1^, Meiqian Xu^1^, Hongliang Yu^1^, Liping Wang^1^, Xiaoxia Li^4^, Janusz Rak^3^, Shihua Wang^1*^ and Robert Chunhua Zhao^1,2*^

^1^Institute of Basic Medical Sciences Chinese Academy of Medical Sciences，School of Basic Medicine Peking Union Medical College

^2^ Department of Cell Biology, School of Life Sciences, Shanghai University, Shanghai 200444, China

^3^Research Institute of the McGill University Health Centre, Glen Site, McGill University, Montreal, Quebec H4A 3J1, Canada.

^4^Department of Genetics and Cell Biology, Basic medical college, Qingdao University, 308 Ningxia Road, Qingdao 266071, China. 266071

Correspondence to: wangshihua@ibms.pumc.edu.cn or zhaochunhua@ibms.pumc.edu.cn

This PDF file includes:

Figures. S1 to S4

Tables. S1 to S2

Figure. S1.


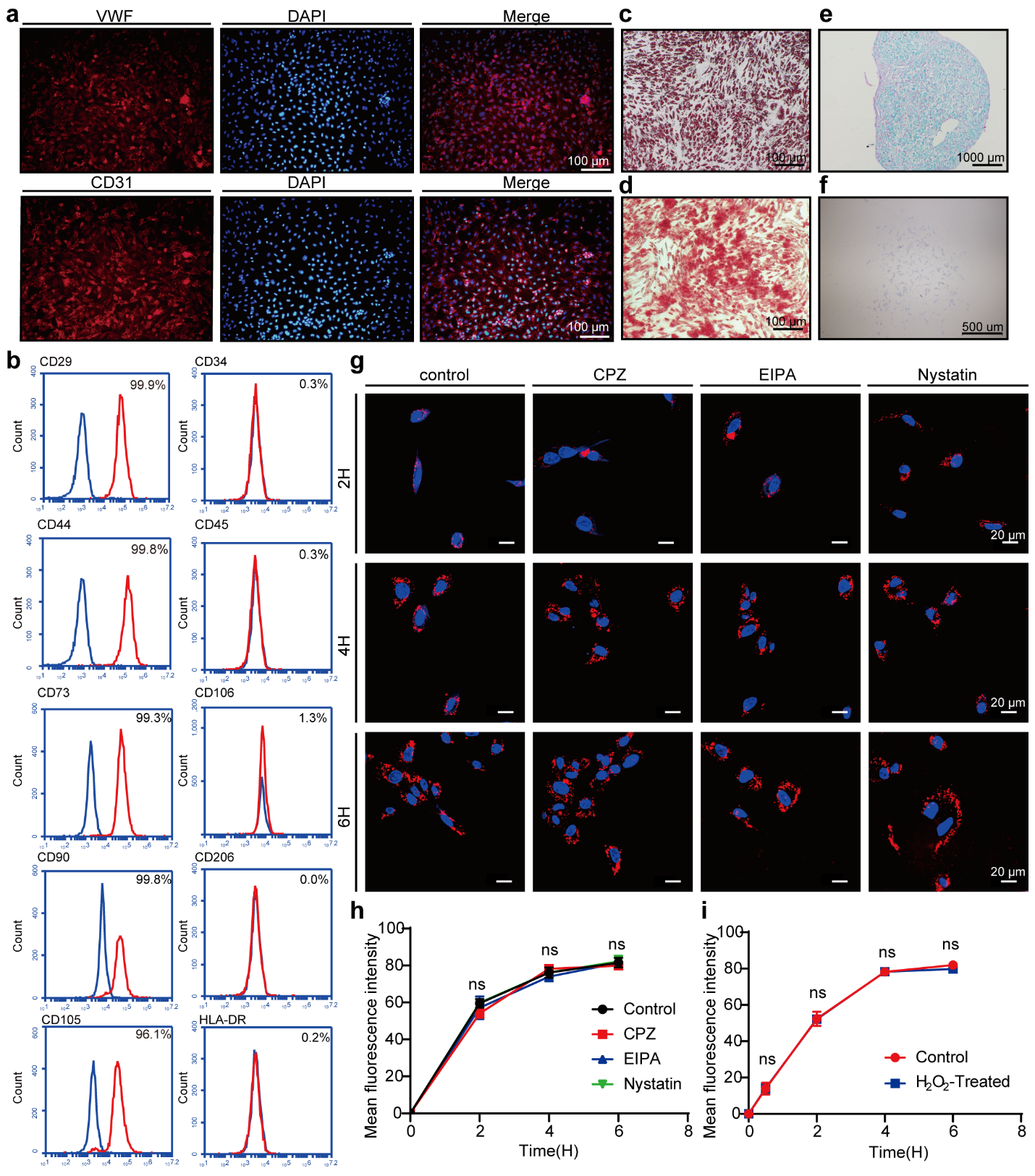


Figure S1. Characterization of HUVECs, MSCs and MSC-sEV internalization by HUVECs.

(a), Representative images of endothelial cells marker VWF and CD31 in isolated HUVECs were determined by fluorescence microscopy (scale bar,100 μm).

(b), Flow cytometry analysis found MSC markers CD29, CD44, CD73, CD90, CD105 are positive, and CD34, CD45, CD106, CD206, HLA-DR are negative.

(c), MSC adipogenic differentiation was demonstrated by Oil-red O staining (scale bar,100 μm). (d), MSC osteogenic differentiation was demonstrated by Alizarin Red staining (scale bar, 100 μm).

(e), Identification of MSC chondrogenic differentiation by Alcian Blue Staining.

(f), Representative image of MSCs colony (scale bar, 500 μm).

(g), Uptake of MSC-sEV by HUVEC pretreated with extracellular vesicle internalization inhibitors CPZ, nystatin, or EIPA was shown by confocal microscope (scale bar, 20 μm).

(h), Fluorescence intensity analysis of MSC-sEV uptaken by HUVEC pretreated with internalization inhibitors CPZ, nystatin, or EIPA, n = 3.

(i), Fluorescence intensity analysis of MSC-sEV uptake by HUVEC pretreated with or without H_2_O_2_ (50 μM, 2 H), n = 3.

Figure. S2.


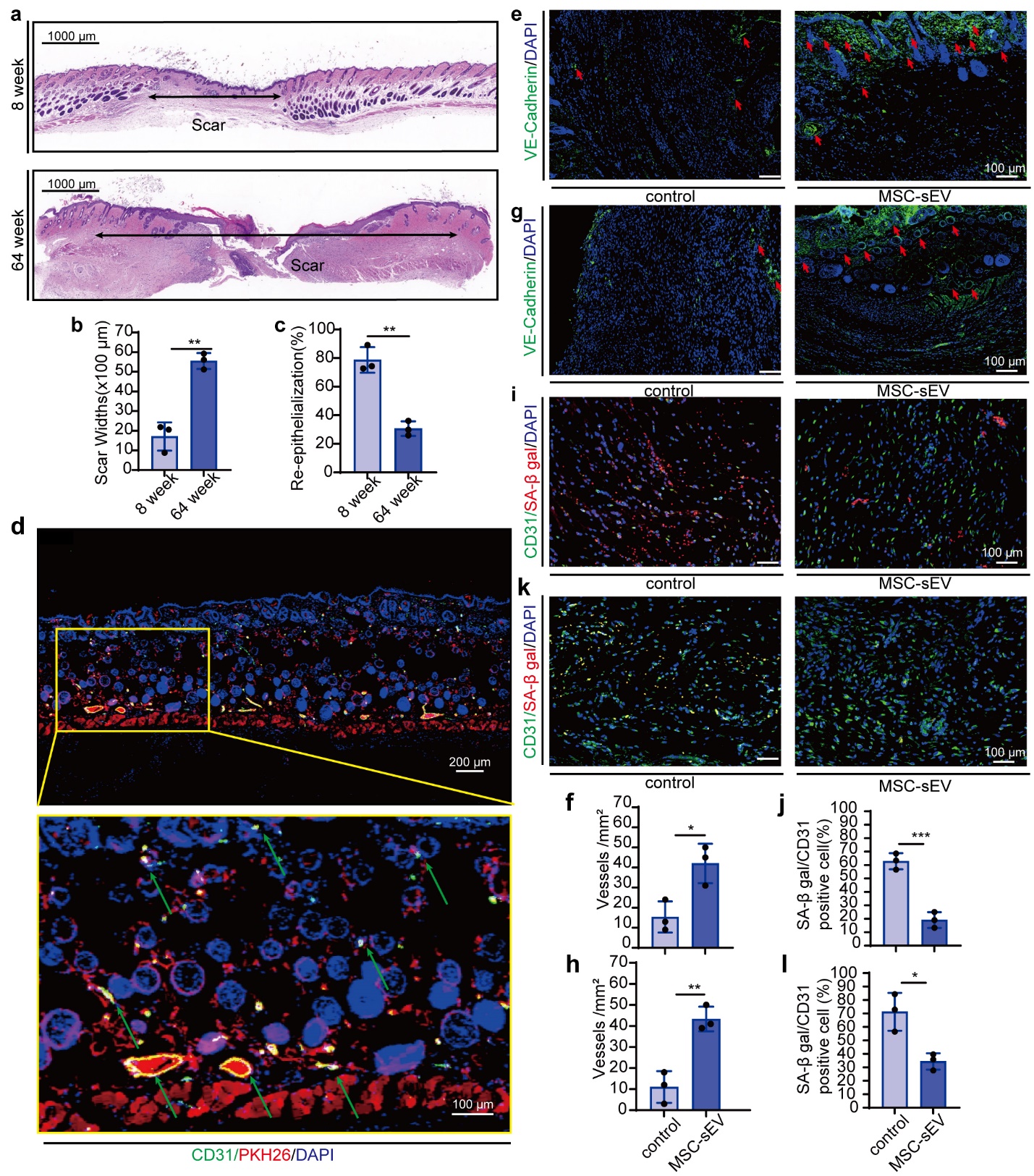


Figure S2. MSC-sEV promoted blood vessel formation attenuated EC senescence in natural aging and type-2 diabetes mice models.

(a), H&E staining of the skin tissue around the wound at day 12 after the operation (horizontal arrows indicated the scar width; scale bar, 1 mm).

(b), Quantification of the scar width around the wound. n = 3, *******p < 0.01*.

(c), Percentage of re-epithelialization around the wound. n = 3, *******p < 0.01*.

(d), Representative immunofluorescence staining images of CD31 positive cells (green staining) and DAPI in paraffin-embedded sections of normal C57BL/6mice dorsal skin injected with PKH26-marked MSC-sEV (red staining). Green arrows indicate uptake of PKH26-marked MSC-sEV by EC (scale bar, 200 μm).

(e), Representative immunofluorescence staining images of VE-cadherin positive cells (green staining) and DAPI in paraffin-embedded sections of aged C57BL/6mice dorsal skin injected PBS (control) or MSC-sEV. Red arrows indicate vessels formed around the wound (scale bar, 100 μm).

(f), Quantification of vessels formed around the scar. n = 3, ******p < 0.05*.

(g), Representative immunofluorescence staining images positive cells of VE-cadherin (green staining) and DAPI in paraffin-embedded sections of diabetic C57BL/6mice dorsal skin injected with PBS (control) or MSC-sEV. Red arrows indicate vessels formed around the wound (scale bar, 100 μm).

(h), Quantification of vessels formed around the scar. n = 3, *******p < 0.01*.

(i), Representative immunofluorescence staining images of SA b-gal (red staining), and CD31-positive cells (green staining) on paraffin-embedded sections of aged model C57BL/6mice dorsal skin injected with PBS (control) or MSC-sEV (scale bar, 50 μm).

(j), Quantitation of SA b-gal positive cells in CD31-positive cells. n = 3, ********p < 0.001*.

(k), Representative immunofluorescence staining images of SA b-gal (red staining), and CD31-positive cells (green staining) on paraffin-embedded sections of type-2 diabetes model C57BL/6mice dorsal skin injected with PBS (control) or MSC-sEV (scale bar, 50 μm).

(l), Quantitation of SA b-gal positive cells in CD31-positive cells. n = 3, ******p < 0.05*.

Figure. S3.


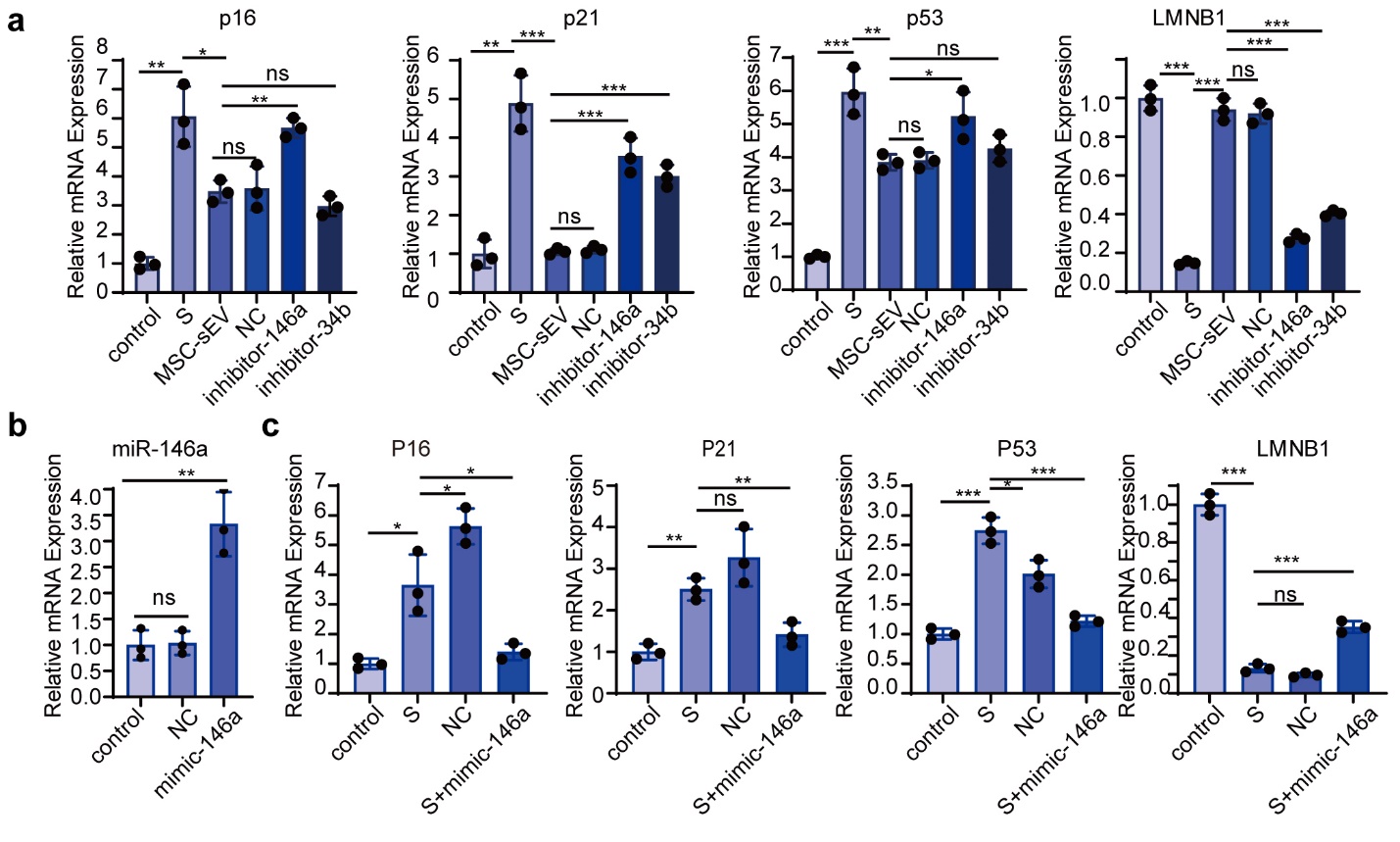


Figure S3. Characterization of transfection of inhibitor-146a, inhibitor-34b, and mimic-146a in HUVECs.

(a), qRT-PCR of miRNA-146a in HUVECs after transfection. n = 3, ******p < 0.05*, *******p < 0.01*, ********p<0.001*.

(b), qRT-PCR of the senescence markers P16, P21, P53 and LMNB1 mRNA in HUVEC. n = 3, *******p < 0.01*.

(c), qRT-PCR of the senescence markers P16, P21, P53 and LMNB1 mRNA in HUVECs. n = 3, ******p < 0.05*, *******p < 0.01*, ********p<0.001*.

Figure S4.


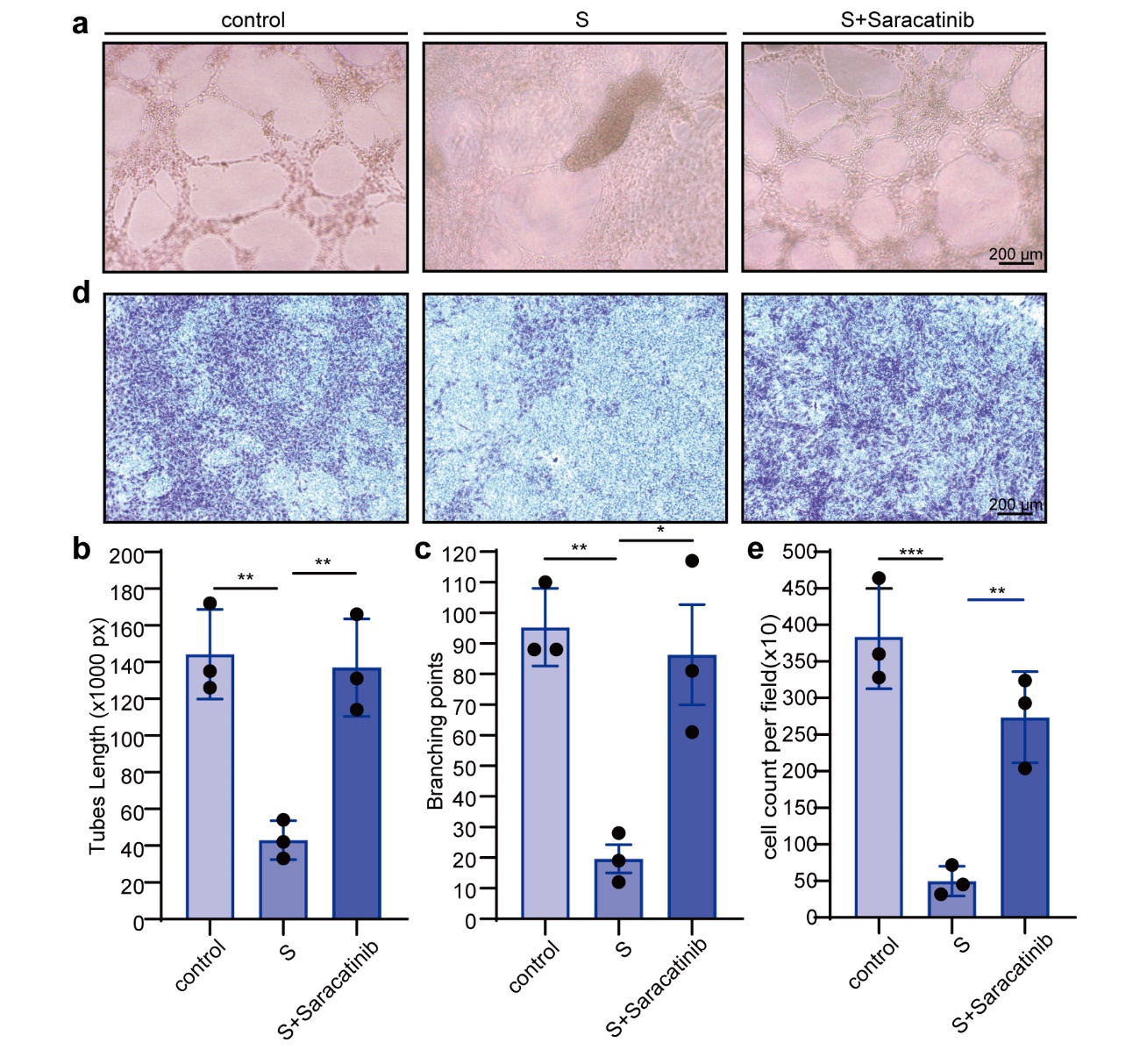


Figure S4. Inhibition of Src rescued senescent HUVECs functions.

(a), Representative images of in vitro tube formation assay (scale bar, 200 μm).

(b), Quantitation of mean tube lengths in the HUVEC network. n = 3, *******p < 0.01*.

(c), Quantitation of branching points in the HUVEC network. n = 3, ******p < 0.05*, *******p < 0.01*.

(d), Representative images of transwell migration assays of HUVECs (scale bar, 200 μm).

(e), Quantitation of transwell migration assays of HUVECs. n = 3, *******p < 0.01*, ********p<0.001*.

Table. S1. Sequences of miRNAs inhibitors, mimics, primers

| Sequences of miRNAs inhibitors, mimics | |  |
| --- | --- | --- |
| Name | **Sequences** | |
| hsa-miR-146a-5p inhibitor  hsa-miR-146a-5p mimic  hsa-miR-34b-3p inhibitor | TGAGAACTGAATTCCATGGGTT  UGAGAACUGAAUUCCAUGGGUU  CAAUCACUAACUCCACUGCCAU | |

| Sequences of RT-qPCR primers |  |
| --- | --- |
| Name | **Sequences** |
| P53-F1 | CAGCACATGACGGAGGTTGT |
| P53-R1 | TCATCCAAATACTCCACACGC |
| P16-F1 | GATCCAGGTGGGTAGAAGGTC |
| P16-R1 | CCCCTGCAAACTTCGTCCT |
| P21-F1 | TGTCCGTCAGAACCCATGC |
| P21-R1 | AAAGTCGAAGTTCCATCGCTC |
| LMNB1-F | AAGCATGAAACGCGCTTGG |
| LMNB1-R | AGTTTGGCATGGTAAGTCTGC |
| PPIA-F | GTCAACCCCACCGTGTTCTT |
| PPIA-R | CTGCTGTCTTTGGGACCTTGT |
| GAPDH-F | TCAACGACCACTTTGTCAAGCTCA |
| GAPDH-R | GCTGGTGGTCCAGGGGTCTTACT |
| hsa-mir-146a-5p F | CGCGTGAGAACTGAATTCCA |
| hsa-mir-146a-5p R | AGTGCAGGGTCCGAGGTATT |
| hsa-miR-28-3p F | CGCGCACTAGATTGTGAGCT |
| hsa-miR-28-3p R | AGTGCAGGGTCCGAGGTATT |
| hsa-miR-412-5p F | CGTGGTCGACCAGTTGGAA |
| hsa-miR-412-5p R | AGTGCAGGGTCCGAGGTATT |
| hsa-miR-34b-3p F | CGCGCAATCACTAACTCCAC |
| hsa-miR-34b-3p R | AGTGCAGGGTCCGAGGTATT |
| RNU6-1F | GCTCGCTTCGGCAGCACATATAC |
| RNU6-1R | CGAATTTGCGTGTCATCCTTGCG |

Table. S2. Antibody and Reagent

| **Antibody** | **Source** | **Identifier** |
| --- | --- | --- |
| P16 | Proteintech | 10883-1-AP |
| P21 | Proteintech | 10355-1-AP |
| P53 | Proteintech | 10442-1-AP |
| LMNB1 | Proteintech | 12987-1-AP |
| p-AKT | CST | 4060T |
| p-ERK1/2 | Abcam | ab76299 |
| p-Src | CST | 4060T |
| p-c-Jun | CST | 2361S |
| p-VE cadherin | Abcam | ab119785 |
| p-Caveolin-1 | Abcam | ab38468 |
| p-P38 | Abcam | ab4822 |
| p-JNK | CST | 4669T |
| JNK | CST | 9252T |
| P38 | Proteintech | 14064-1-AP |
| caveolin-1 | Abcam | ab2910 |
| VE cadherin | Abcam | ab33618 |
| c-Jun | CST | 2361S |
| Src | CST | 2109T |
| AKT | Proteintech | 60203-2-Ig |
| ERK1/2 | proteintech | 16443-1-AP |
| CD63 | Proteintech | 25682-1-AP |
| Calnexin | CST | 2433S |
| TSG101 | Abcam | ab83 |
| Alix | CST | 92880S |
| VWF | Abcam | ab6994 |
| CD31 | Abcam | ab9498 |
| GAPDH | Proteintech | 10494-1-AP  60004-1-Ig |
| HRP-conjugated anti-rabbit-IgG | NeoBioscience |  |
| HRP-conjugated anti-mouse-IgG | NeoBioscience |  |
| **Reagent** | **Source** | **Identifier** |
| Hoechst | Sigma-Aldrich | 33342 |
| Matrigel | BD Biosciences | 354230 |
| ELISA Kit IL-1alpha | R&D | DLA50 |
| ELISA Kit IL-6 | R&D | 550799 |
| ELISA Kit IL-8 | R&D | DY208 |
| Mito Stress Test Kit | Agilent | 103015-100 |
| Fluorometric Intracellular ROS Kit | Sigma Aldrich | MAK144 |
| BCA Protein Assay Kit | Beyotime | \|  \| P0011 \| \| --- \| --- \| |
| TRIzol | Invitrogen | 15596018 |
| Lipo2000 | Invitrogen | 11668-019 |
| STZ | Sigma-Aldrich | S0130 |
| MTS | Promega | G3581 |
