# Supplementary figures and images for: Correction to: Mesenchymal stem cell-derived small extracellular vesicles mitigate oxidative stress-induced senescence in endothelial cells via regulation of miR-146a/Src
Source: Signal Transduct Target Ther. 2022 Jul 14;7:234. doi: 10.1038/s41392-022-01075-y (PMC9283623; doi:10.1038/s41392-022-01075-y)

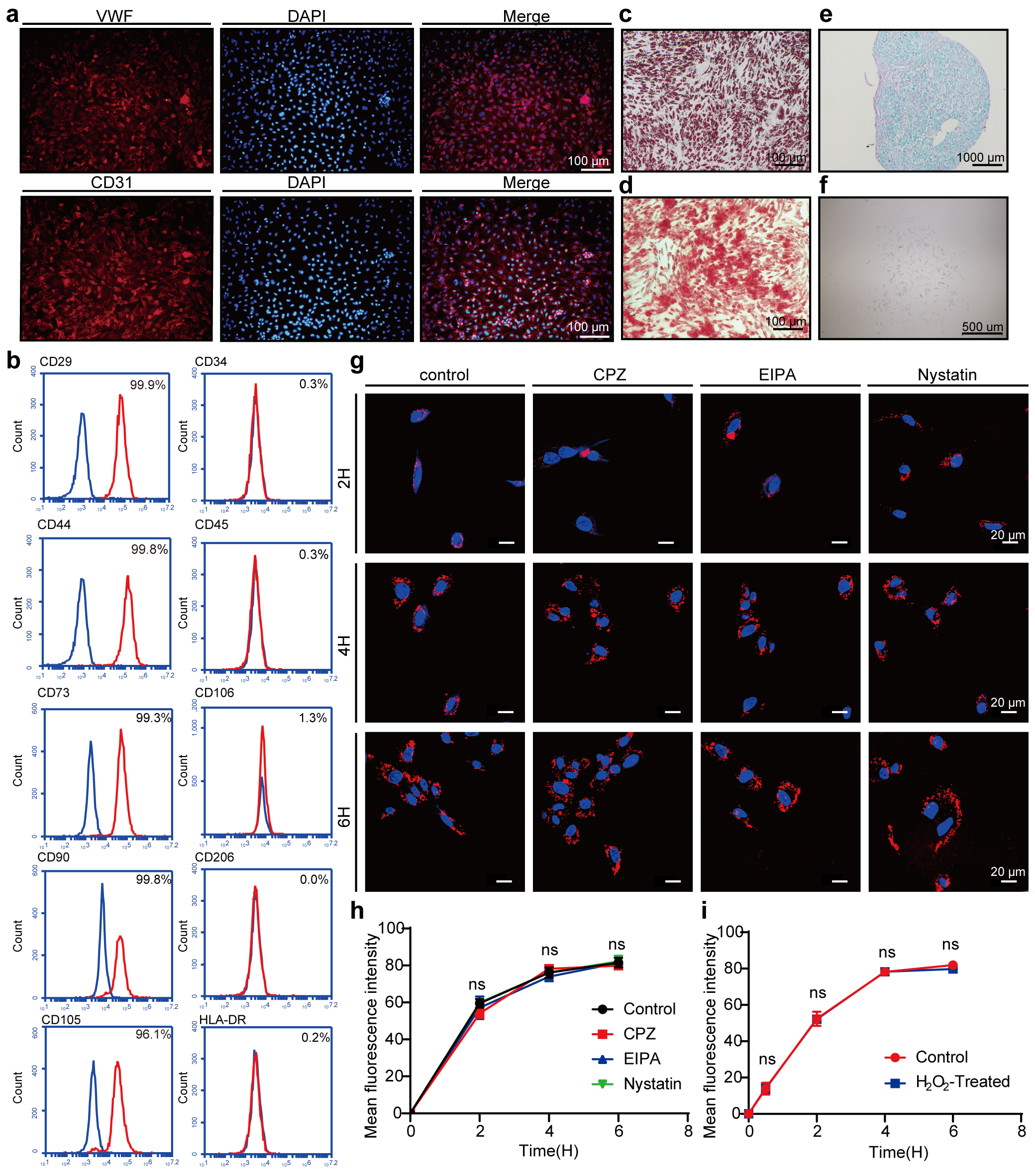

Supplement: Supplementary file 2 — Revised Supplementary figure 1 [file 41392_2022_1075_MOESM2_ESM.tif]
